# Co-occurring homelessness, justice involvement, opioid dependence and psychosis: a cross-sectoral data linkage study

**DOI:** 10.1093/eurpub/ckad034

**Published:** 2023-03-15

**Authors:** Emily J Tweed, Alastair H Leyland, David S Morrison, S Vittal Katikireddi

**Affiliations:** MRC/CSO Social and Public Health Sciences Unit, University of Glasgow, Glasgow, UK; MRC/CSO Social and Public Health Sciences Unit, University of Glasgow, Glasgow, UK; Institute of Health and Wellbeing, University of Glasgow, Glasgow, UK; MRC/CSO Social and Public Health Sciences Unit, University of Glasgow, Glasgow, UK

## Abstract

**Background:**

Administrative data offer unique opportunities for researching experiences which pose barriers to participation in primary research and household surveys. Experiencing multiple social disadvantages is associated with very poor health outcomes, but little is known about how often this occurs and what combinations are most common. We linked administrative data across public services to create a novel population cohort containing information on experiences of homelessness, justice involvement, opioid dependence and psychosis.

**Methods:**

We securely linked administrative data from (i) a population register derived from general practitioner registrations; (ii) local authority homelessness applications; (iii) prison records; (iv) criminal justice social work reports; (v) community dispensing for opioid substitution therapy; and (vi) a psychosis clinical register, for people aged ≥18 years resident in Glasgow, Scotland between 01 April 2010 and 31 March 2014. We estimated period prevalence and compared demographic characteristics for different combinations.

**Results:**

Of 536 653 individuals in the cohort, 28 112 (5.2%) had at least one of the experiences of interest during the study period and 5178 (1.0%) had more than one. Prevalence of individual experiences varied from 2.4% (homelessness) to 0.7% (psychosis). The proportion of people with multiple co-occurring experiences was highest for imprisonment (50%) and lowest for psychosis (14%). Most combinations showed a predominance of men living in the most deprived areas of Scotland.

**Conclusions:**

Cross-sectoral record linkage to study multiple forms of social disadvantage showed that co-occurrence of these experiences was relatively common. Following this demonstration of feasibility, these methods offer opportunities for evaluating the health impacts of policy and service change.

## Introduction

Administrative data generated by organizations as part of routine activities are increasingly used in research, thanks to potential time and cost savings; broad socio-demographic, geographical and temporal coverage; and high external validity and policy relevance.^1^ Linkage of administrative datasets is especially valuable to social epidemiology, given the diversity of factors which influence health at the individual, community, environmental and societal level.^2^^,^^3^ Such linkages offer the possibility of ‘real-world’ evidence able to inform policy making across multiple sectors to improve population health and reduce health inequalities, an endeavour often referred to as healthy public policy.^3^^,^^4^

These methodological developments are especially relevant to understanding the needs of population groups who experience marginalization and disadvantage, who are often under-represented in primary research.^5^ For instance, they may find it difficult to participate in cohort studies requiring active follow-up, leading to threats to validity from non-participation and attrition biases.^6^^,^^7^ However, they are often well-represented in administrative datasets due to high levels of need for, and utilization of, public services.^8^ Administrative data also enable analysis of relatively rare exposures and outcomes thanks to large population sizes not attainable through direct recruitment.^1^

We sought to understand the feasibility and value of linked administrative data in this context through a proof-of-principle study examining overlapping experiences of homelessness, criminal justice involvement, opioid dependence and psychosis. These experiences were selected as ‘sentinel’ experiences of marginalization and disadvantage which: are of major policy interest in high-income countries (and in some cases increasing in prevalence); are associated with profound inequalities in morbidity and mortality; and appear to commonly co-occur.^9–12^

Responses to these experiences often consider each in isolation, resulting in fragmented services or conflicting goals.^9^^,^^13^^,^^14^ Understanding their intersection is therefore critical for effective policy and service design. Since the extent of this intersection will depend on contextual factors such as healthcare provision, welfare regimes and housing markets, informed public policy requires national and sub-national estimates.^15^^,^^16^

Here, we describe the use of record linkage between multiple administrative datasets to create and characterize a population-based cohort including information on exposure to homelessness, justice involvement, opioid dependence and/or psychosis, as a baseline for subsequent longitudinal studies.

## Methods

### Study design and setting

We undertook a retrospective cohort study using cross-sectoral record linkage between six administrative datasets. We chose Glasgow City local authority in the west of Scotland as our geographical setting, based on the feasibility of data access and local policy interest in co-occurring disadvantage. Glasgow City is an urban area with a population of just over half a million people, representing 11% of the population of Scotland.^17^ The study period for the primary analysis was chosen as 1 April 2010 to 31 March 2014 to maximize availability and quality of study datasets: sensitivity analyses varying this period are described below under ‘Statistical analysis’.

### Population

We obtained data on individuals resident in Glasgow City using the NHS Greater Glasgow and Clyde (NHSGGC) population register, selected on postcode of residence. This dataset is derived from general practitioner registrations and is widely used in record linkage studies as a proxy for total population. It is updated with information on deaths or migration out of the NHSGGC area (within which Glasgow City lies; changes of residence within the health board, including between local authorities, are not recorded). Exclusion criteria were any of:

- Record of having died or moved out of NHSGGC prior to end of study period.
- Aged <18 years or ≥75 years at the start of the study period.
- Turned 75 years of age during study period.

### Experiences of interest

To identify individuals assessed as homeless or threatened with homelessness (see Supplementary material for definitions) we used HL1, a statutory data collection on people seeking statutory homelessness support which is mandatory for all Scottish local authorities.^18–20^

Data on justice involvement were obtained from two sources. Records of individuals received into prisons across Scotland, whether sentenced or on remand, were obtained using the PR2 dataset, a record-keeping system used by all Scottish prisons.^21^ Criminal justice social work report (CJSWR) data were used to identify people convicted of an offence undergoing social work assessment by Glasgow City Council during the study period: details of criteria for reports are detailed in the Supplementary material. Since imprisonment is mutually exclusive with other exposures, and differs substantially in lived experience to community justice involvement, we classified justice involvement on a hierarchical basis using two categories: any experience of prison custody regardless of whether a court report was available (hereafter abbreviated to CUST) or community justice experience only (COMM; i.e. court report without imprisonment).

Data from the Prescribing Information System (PIS), which records dispensing events at community pharmacies across Scotland, were used to identify individuals who had received opioid substitution therapy (OST) in the community as treatment for opioid dependence (hereafter abbreviated as ODep).^22^

Data on individuals with a diagnosis of primary psychotic disorder (see Supplementary material for ICD-10 codes) were identified from the Glasgow Psychosis Clinical Information System (PsyCIS), a clinical registry of demographic, social and treatment data collected from both administrative records and active follow-up.^23^

Further information on the datasets can be found in the Supplementary section S6.1.

All of the study datasets have previously been used for health research, including through record linkage, with the exception of CJSWR.^19^^,^^21^^,^^22^^,^^24^

We defined exposure to each of the experiences of interest as at least one appearance in the relevant dataset during the study period: combinations reported here therefore reflect cumulative exposure across the study period. For clarity of reporting, we describe the prevalence of all potential exposure combinations, but where describing demographic characteristics, use a simpler two-category approach comprising each experience in isolation or in combination, e.g. homelessness only vs. homelessness + other experience(s).

### Covariates

All analyses used demographic characteristics as recorded in the population register. The exception to this was ethnicity, which was only recorded in HL1, PR2, CJSWR and PsyCIS datasets and is therefore only reported for these sources (see Supplementary material). Age was calculated at the end of the study period, given that this was the point at which cumulative exposure was measured. The Scottish Index of Multiple Deprivation (2012 release) was used to approximate socioeconomic circumstances, based on postcode of residence.^25^

### Data sharing and access

The Local Privacy Advisory Committee of the West of Scotland Safe Haven provided approval to access data from the NHSGGC population register, PIS, and PsyCIS, and support with data linkage and storage. The Data Protection Officer and relevant Head of Service of Glasgow City Council and Health and Social Care Partnership granted access to HL1 and CJSWR datasets. The Scottish Government Statistics Public Benefit and Privacy Panel and Scottish Prison Service Research Access and Ethics Committee granted access to the PR2 dataset. The study was also approved by the University of Glasgow College of Medical, Veterinary, and Life Sciences research ethics committee.

### Record linkage

**Figure 1:**
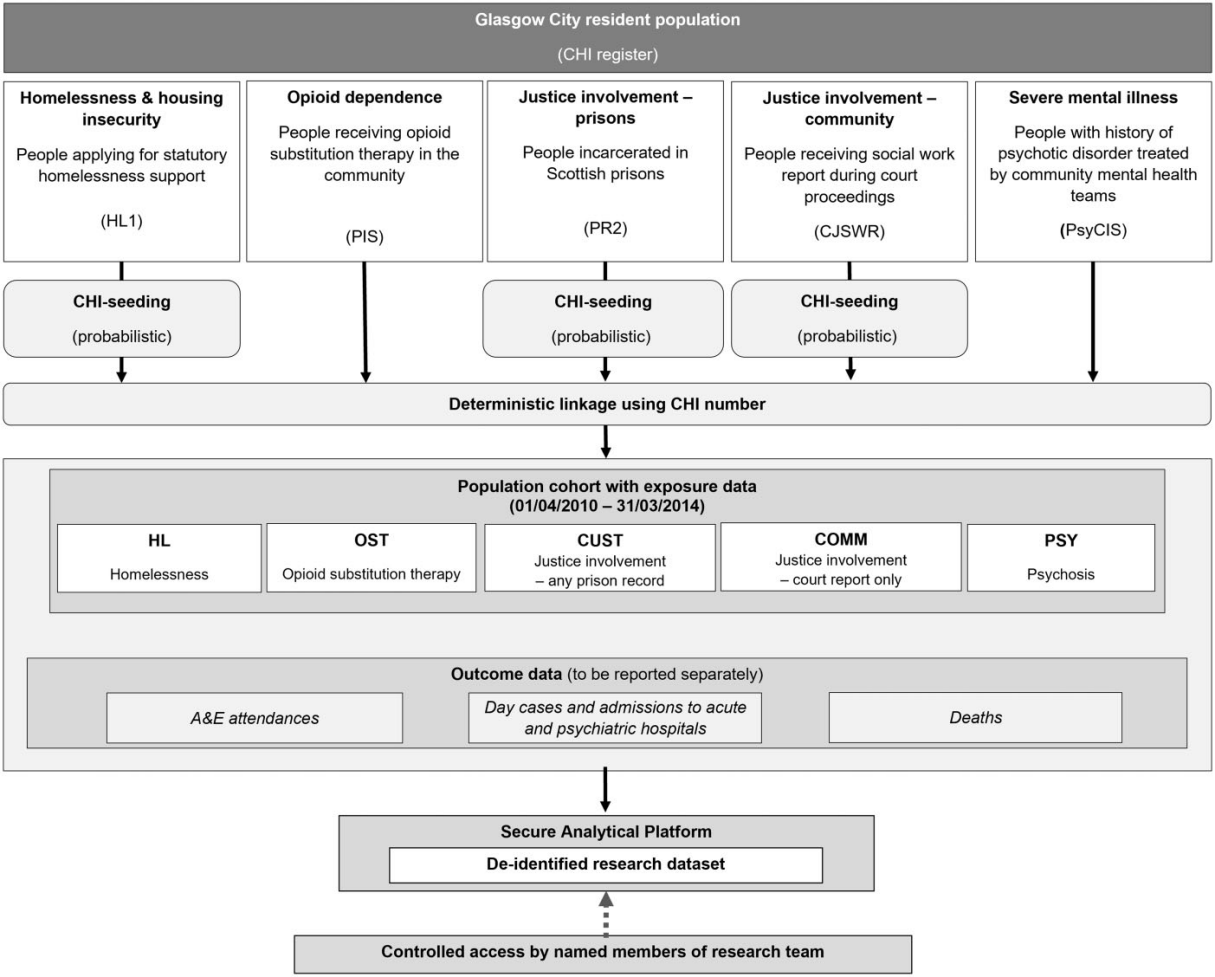
Schematic diagram illustrating linkage process for creation of cohort

Record linkage between datasets was undertaken by the West of Scotland Safe Haven using the Community Health Index (CHI) number, a unique 10-digit numeric identifier used across the health service in Scotland.^26^ Of the exposure data sources, PIS and PsyCIS already contained CHI numbers for all individuals; CJSWR contained CHI for some. CHI numbers were identified for individuals in HL1, PR2 and the remaining individuals in CJSWR by matching to the population register using forename, surname, date of birth and postcode (see Supplementary material). The same exclusion criteria were applied to the exposure datasets as to the population register (i.e. individuals were excluded if they were recorded in the population register as having died or moved out of NHSGGC prior to end of the study period; were aged <18 years or ≥75 years at the start of the study period; or turned 75 years of age during the study period). Following linkage, de-identified data were accessed by the research team for analysis via a secure analytic platform. The linkage process is illustrated in figure 1.

### Statistical analysis

Data were cleaned and analysed in Stata 16 (StataCorp, TX, USA), with visualizations created with R version 4.0.3 using *ggplot2*.

We used descriptive statistics to investigate the association between exposure combinations and demographic characteristics, and created UpSet plots—an alternative to Venn diagrams for >3 sets—to visualize intersections between the experiences of interest.^27^ We undertook sensitivity analyses of study period length on the prevalence and intersection of the exposures of interest. We also undertook sensitivity analyses of our primary estimate of period prevalence using Census-derived mid-year population estimates as the denominator, rather than the population register.

### Public and stakeholder involvement

Analyses and interpretation were informed by public engagement workshops with people with lived/living experience of the issues of interest and a series of advisory group meetings including stakeholders from academia, NHS, local government, Scottish Government and the third sector, and two further public representatives.

## Results

In undertaking the linkage, the three datasets from non-health sources (HL1, PR2 and CJSWR) varied in the proportion of records which could be matched to the population register (Supplementary material). CHI was already known for a high proportion of records within the CJSWR dataset, so overall only 8% of records could not be assigned a CHI number and there was little difference in age and gender profile between records for which a CHI number could and could not be identified. In contrast, for homelessness (HL1) and prisons (PR2) datasets, 22% and 24% of records respectively could not be assigned a CHI number. For both the HL1 and PR2 datasets, the mean age was similar for records which could and could not be matched but the proportion of men among the former was slightly higher.

The final cohort comprised 536 653 unique adults resident in Glasgow City who were alive at the end of the study period on 31 March 2014 (table 1).

**Table 1:** Prevalence of experiences of disadvantage among adults living in Glasgow City, 01 April 2010–31 March 2014

Experience(s) of interest	Number of individuals	Percentage of total cohort, i.e. period prevalence (%)	Percentage of exposed cohort (%)
Total population	536 653	100.00	–
No experiences of interest	508 541	94.8	–
Any experience of interest	28 112	5.2	100.0
Summary of combinations^a^			
Any homelessness (HL)	13 075	2.4	46.5
HL only	9463	1.8	33.7
HL + other experience(s)	3612	0.7	12.9
Any opioid dependence (ODep)	7412	1.4	26.4
ODep only	4123	0.8	14.7
ODep + other experience(s)	3289	0.6	11.7
Any justice involvement—custodial (CUST)	5512	1.0	19.6
CUST only	2755	0.5	9.8
CUST + other experience(s)	2757	0.5	9.8
Any justice involvement—community (COMM)	4619	0.9	16.4
COMM only	3338	0.6	11.9
COMM + other experience(s)	1281	0.2	4.6
Any psychosis (PSY)	3791	0.7	13.5
PSY only	3255	0.6	11.6
PSY + other experience(s)	536	0.1	1.9
Detailed combinations: mutually exclusive categories^b^			
Homelessness (HL) only	9463	1.8	33.7
Opioid dependence (ODep) only	4123	0.8	14.7
Justice—community (COMM) only	3338	0.6	11.9
Psychosis (PSY) only	3255	0.6	11.6
Justice—custodial (CUST) only	2755	0.5	9.8
HL + CUST	994	0.2	3.5
ODep + CUST	846	0.2	3.0
HL + ODep	820	0.2	2.9
HL + ODep + CUST	780	0.2	2.8
HL + COMM	574	0.1	2.0
ODep + COMM	433	0.1	1.5
HL + ODep + COMM	195	<0.1	0.7
HL + PSY	159	<0.1	0.6
ODep + PSY	135	<0.1	0.5
PSY + CUST	61	<0.1	0.2
PSY + COMM	56	<0.1	0.2
HL + PSY + CUST	35	<0.1	0.1
HL + PSY + ODep	26	<0.1	0.1
ODep + PSY + CUST	25	<0.1	0.1
HL + ODep + PSY + any justice involvement^c^	19	<0.1	0.1
HL + PSY + COMM	10	<0.1	<0.1
ODep + PSY + COMM	10	<0.1	<0.1

a Ordered by frequency of ‘any’ category.

b Ordered by frequency of mutually exclusive categories.

c Results for HL + ODep + PSY + COMM and HL + ODep + PSY + CUST are grouped here due to small numbers, to avoid presenting potentially disclosive information.

Considering each exposure in isolation, between 01 April 2010 and 31 March 2014, a total of 13 075 (2.4%) people were assessed as homeless or threatened with homelessness at least once; 5512 (1.0%) were received into prison at least once; 7954 (1.5%) had at least one criminal justice social work report; 7412 (1.4%) had at least one episode of OST dispensing; and 3791 (0.7%) appeared in the psychosis case register. In total, 28 112 (5.2%) people had one or more of the experiences of interest. Of 7954 individuals with a criminal justice social work report, 3335 (41.9%) also experienced imprisonment (hereafter referred to as CUST), leaving 4619 (58.1%) in contact with community justice without any imprisonment during the study period (COMM).

**Figure 2:**
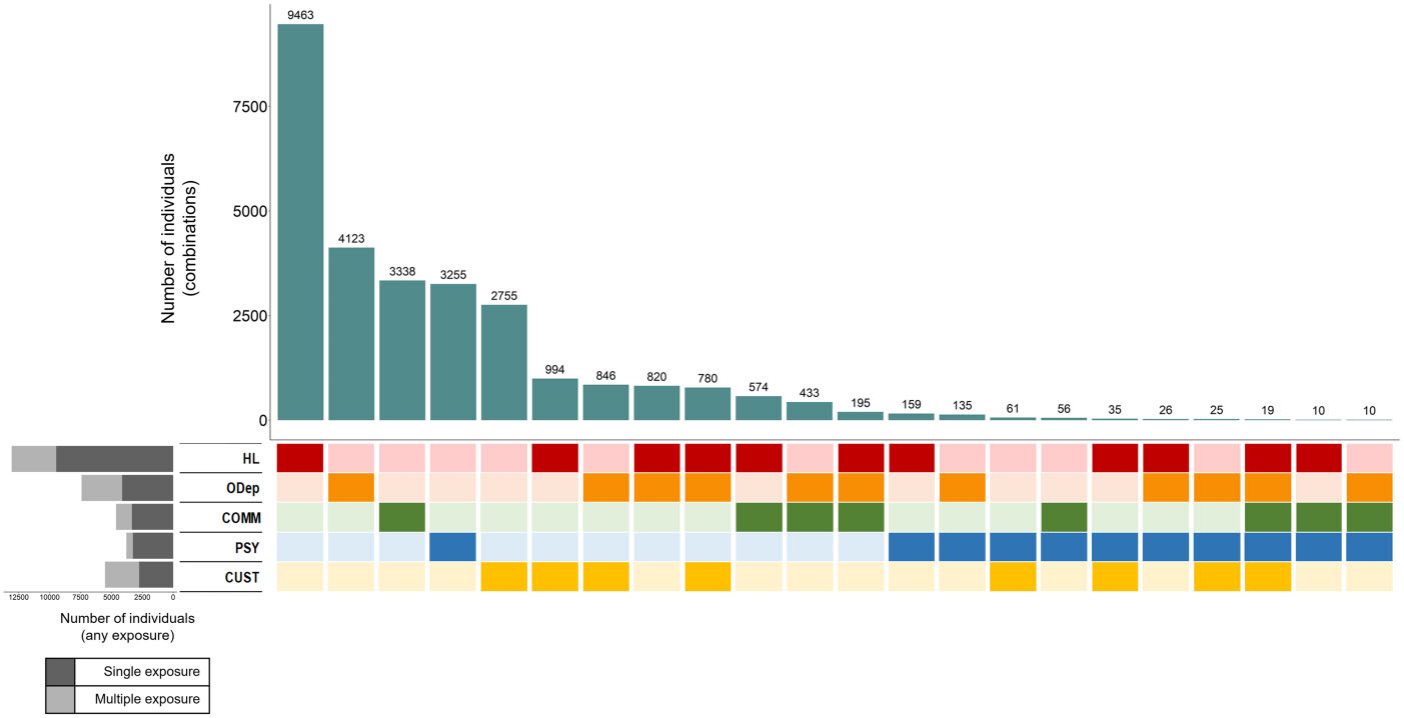
UpSet plot showing frequency of mutually exclusive exposure combinations (vertical bars) and any exposure (horizontal bars) among adults living in Glasgow City, 01 April 2010–31 March 2014

The most common combinations were those involving homelessness, opioid dependence, and justice involvement; combinations involving psychosis were much less common (figure 2). Across the cohort, 5178 people (1.0% of the cohort) had more than one exposure, though numbers of those with three or more were small (table 1). Within each exposure, the proportion with or without additional exposures varied (figure 2; Supplementary table S6.3.1): co-occurrence was highest among people who had been in prison (50%, *n* = 2757/5512) and lowest among people with psychosis (14%, *n* = 536/3791). Overlaps between exposure pairs are shown in Supplementary material.

People imprisoned during the study period—the only experience mutually exclusive with the others—spent a median total of 130 days (9% of the 4-year study period) in prison, with 77% (*n* = 1266/5512) incarcerated for <1 year in total (Supplementary material). The distribution of total prison time was similar between people imprisoned who did and did not experience any of the other exposures (Supplementary material).

**Figure 3:**
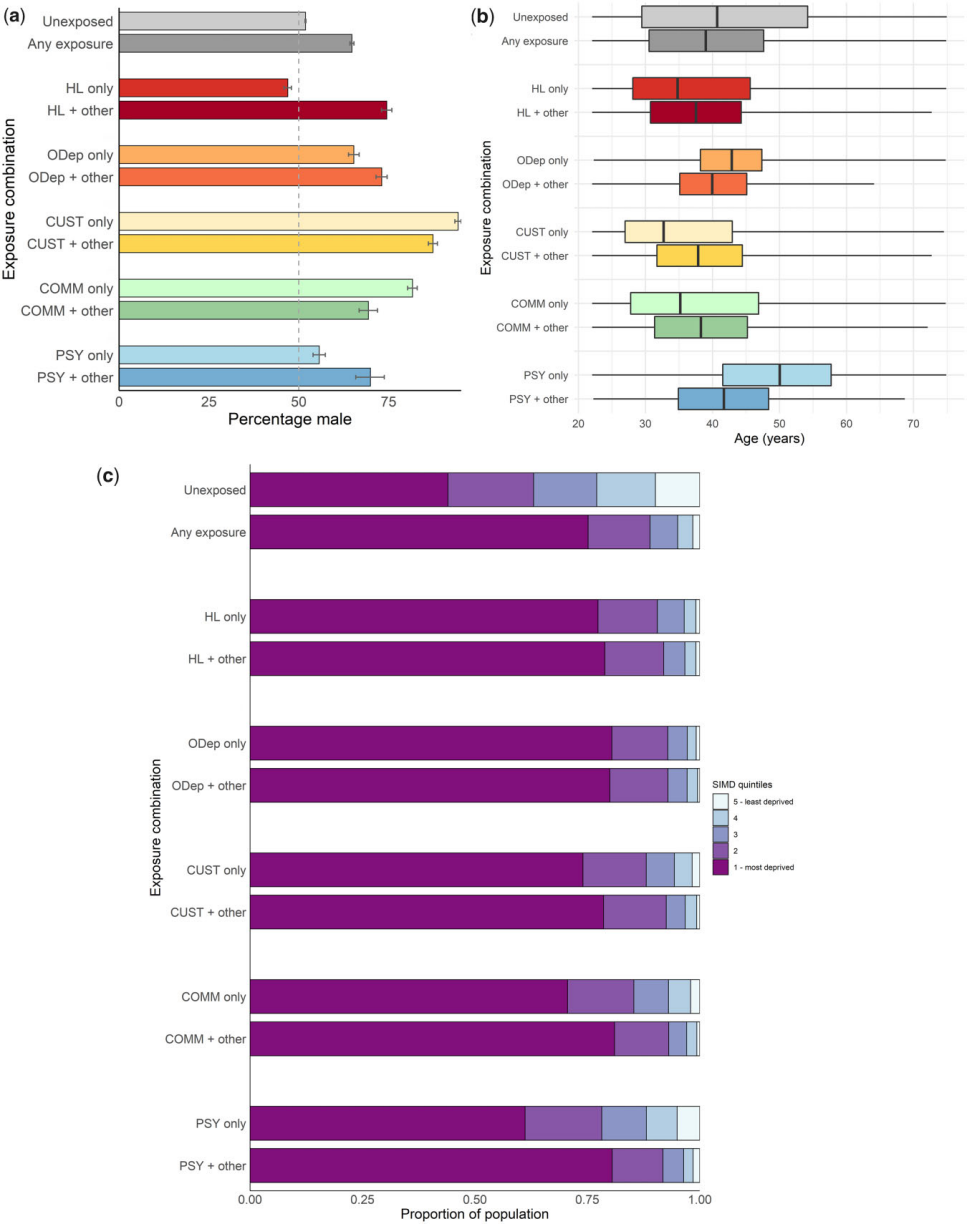
(a) Percentage male (with 95% confidence intervals) among adults living in Glasgow City, stratified by exposure to experiences of disadvantage (01 April 2010–31 March 2014). (b) Boxplot of age distribution among adults living in Glasgow City, stratified by exposure to experiences of disadvantage (01 April 2010–31 March 2014). (c) Scottish Index of Multiple Deprivation quintile distribution among adults living in Glasgow City, by exposure to experiences of disadvantage (01 April 2010–31 March 2014)

With regard to gender, justice involvement (especially prison) showed the greatest male predominance (figure 3a; Supplementary table S3.1). The only category which did not show a male predominance was homelessness in isolation. People experiencing homelessness or justice involvement tended to be younger than people with opioid dependence or experiencing psychosis; this was also true for combinations involving these experiences (figure 3b, Supplementary table S3.1). There was no consistent association between age and multiple experiences. People with any experience of interest were more likely to live in more deprived areas compared to the unaffected group, though this tendency was less pronounced for psychosis than for other experiences (figure 3c, Supplementary table S3.1). Recorded ethnicity data indicated that the large majority of individuals in the homelessness, prisons, CJSWR, and psychosis datasets were White (Supplementary material).

For experiences consisting of clearly-defined episodes (homelessness, imprisonment and community justice), people with multiple forms of disadvantage tended to have more episodes during the study period than those with only one experience, but these distributions overlapped substantially (Supplementary material).

Table 1 shows estimated period prevalence for the exposures of interest in Glasgow City among the study cohort over the 4-year exposure period. Sensitivity analyses showed that varying the length of the exposure period had the greatest impact on prevalence of combinations involving homelessness, followed by community justice involvement; there was little change in the estimated prevalence of combinations involving psychosis, imprisonment or opioid dependence (Supplementary material). Further sensitivity analyses showed that use of a population denominator derived from census estimates, rather than the population register, slightly increased the estimated prevalence of experiences of interest but did not affect conclusions about relative frequency (Supplementary material).

## Discussion

We have demonstrated the feasibility of a unique cross-sectoral record linkage to create and characterize a cohort of people who, because of their life circumstances, may be less likely to participate in primary research or household surveys. Around 5% of the population experienced any of the five forms of disadvantage of interest during the study period, with 1% of the population affected by more than one. The majority of those experiencing multiple forms of disadvantage were White males aged between 30 and 50 years living in the most socioeconomically deprived areas, but profiles differed between different combinations, with those experiencing psychosis forming a relatively distinct population.

Although the co-occurrence of these experiences is associated with much higher rates of morbidity and mortality compared to one or none,^10^ there are very few population-based estimates of the prevalence and patterning of this phenomenon and none to our knowledge using individual-level record linkage. Almost all previous research draws on samples selected on the basis of at least one ‘index’ exposure and is limited in its reporting of different combinations (e.g. 28, 29, 30). In contrast, we were able to explore the prevalence of each experience singly and in combination across an unselected geographic population of adults.

The ‘Hard Edges’ project has previously attempted to quantify the population overlap of homelessness, offending, and substance use in the UK, by combining weighted estimates from survey and routine data sources.^9^^,^^12^ Our approach, using individual-level linkage across administrative datasets, enabled us to minimize reporting and recall biases associated with self-report, as well as participation biases inherent in household and targeted surveys; it also permits longitudinal analyses of health and social outcomes (to be reported separately). While the definitions and data sources vary somewhat, we corroborate Hard Edges’ findings regarding demographic profile; homelessness as the most common experience overall; and justice involvement as the experience most likely to overlap with others.^9^^,^^12^

This work demonstrates the potential for cross-sectoral administrative data linkage to respond to and inform policy priorities. Our findings are particularly timely given ongoing initiatives in Scotland to expand Housing First services for people with homelessness and other disadvantages; new models of joint working to address internationally high rates of drug-related deaths; and concerns about through care support in prisons.^28^^,^^29^ This cohort also offers rich possibilities for evaluating the health impacts of social policies (such as welfare reform or homelessness prevention), through natural experiment designs and policy decision modelling.

Among this study’s strengths are the novelty and breadth of the cross-sectoral data linkage, creating a large population-based cohort. Ongoing longitudinal analyses will build on the proof-of-principle results reported here by investigating health outcomes among the cohort.

By using administrative data, the cohort is likely to be more comprehensive and representative than would be feasible through primary research. For instance, whilst most homelessness research to date has recruited participants from shelters, soup kitchens or the streets, administrative data can ensure the inclusion of those experiencing less visible forms of homelessness, such as ‘sofa-surfing’. Similarly, using community OST dispensing data will include those treated in primary care as well as in specialist drug services; most previous research has focused on the latter. However, this comprehensive approach does potentially result in greater heterogeneity within each category and we classified experiences on a relatively simple cumulative basis that does not account for their dynamic nature over time.

Ascertainment of these experiences is not complete—for instance, HL1 data only included main applicants rather than all adults in the household, and criminal justice social work reports are not completed for every individual convicted in the courts. Relying on administrative data may miss individuals not engaged with services, though use of up to 6 years of data allowed us to test the sensitivity of our results to length of study period and maximized ascertainment of individuals who may engage on a transient or infrequent basis. In future, our methods may be also strengthened by triangulation between multiple administrative datasets relating to the same experience (e.g. from third sector as well as statutory services); cohorts recruited as part of primary research; and novel means of interrogating existing datasets (e.g. data phenotyping approaches).^30–32^ Future work will also seek to extend the cohort nationally, to capture regional variation and maximize generalizability to other settings.

The CHI register is the best available source of population data in Scotland at present but may omit individuals not registered with primary care or incorrectly include those who have not de-registered after moving out of the area. A proportion of records from the non-health datasets could not be matched to a CHI number and were therefore not included in the cohort. As the CHI register is a live database updated on a regular basis, a failure to match may result from individuals having moved out of the NHSGGC area since being recorded in the exposure dataset or from incorrect identifiers recorded in one or multiple datasets. We are not able to distinguish between these possibilities, or to assess potential impact on representativeness or risk of bias, though the broadly comparable age and gender profile for matched and unmatched populations is reassuring.^33^

Very few previous studies have reported measures of linkage success for comparison.^21^^,^^33–36^ Waugh *et al*.’s^18^ national study using HL1 was able to match more than 90% of records, though had access to a national population spine and a more complex linkage algorithm. Other studies using regional or local datasets from non-health sources report linkage success rates between 80% and 90%, more comparable to those observed here.^19^^,^^37^^,^^38^ Our study used relatively stringent matching criteria compared to those reported elsewhere, which often rely more heavily on probabilistic approaches using score-based thresholds and is therefore likely to have prioritized specificity at the expense of sensitivity. However, in the absence of a gold standard, we are unable to assess these metrics quantitatively. Future work using national datasets, prospective rather than retrospective linkages, and/or sensitivity analyses applying different linkage thresholds may offer opportunities to evaluate and improve linkage success.^39^

As acknowledged above, the occurrence of these experiences is closely linked to policy and service context and therefore likely to vary from area to area. There are relatively few population-based estimates of the co-occurrence of these experiences from other areas to use in assessing generalizability of our findings. The ‘Hard Edges Scotland’ project (which also used a population-based approach, albeit not based on individual-level linkage) found that co-occurring homelessness, justice involvement and problem drug use was highest in urban and poorer areas in Central Scotland like Glasgow City, but also in other major urban centres such as Dundee and Aberdeen.^9^ Glasgow has historically experienced very high concentrations of socioeconomic deprivation and related excess mortality, which may limit generalizability to other areas.^40^ Application of our methods in other settings offers the opportunity to obtain locally-relevant estimates and potentially to undertake comparisons and evaluations of the impact of different policy approaches.

Despite these limitations, our results provide novel insights into a cohort of people in contact with services who may be reached through interventions to prevent or mitigate health and social inequalities. Realizing the potential of cross-sectoral data linkage for informing healthy public policy depends on well-resourced and responsive infrastructure and governance processes. Close collaboration between researchers and other stakeholders is also critical, to understand data availability and provenance, inform interpretation of findings, and identify priorities for further work.

## Supplementary Material

ckad034_Supplementary_Data

## Data Availability

Access to the de-identified study dataset is restricted under the terms of the ethical approvals granted by data controllers. Researchers wishing to re-use the datasets described in this study may apply to the relevant agencies for permission to do so.

## References

[1] Connelly R , Playford CJ, Gayle V, Dibben C. The role of administrative data in the big data revolution in social science research. Soc Sci Res 2016;59:1–12. 27480367 10.1016/j.ssresearch.2016.04.015

[2] Lyons RA , Ford DV, Moore L, Rodgers SE. Use of data linkage to measure the population health effect of non-health-care interventions. Lancet 2014;383:1517–9. 24290768 10.1016/S0140-6736(13)61750-X

[3] Academy of Medical Sciences. Improving the Health of the Public by 2040: Optimising the Research Environment for a Fairer, Healthier Future. London: Academy of Medical Sciences, 2016.

[4] Rutter H , Savona N, Glonti K, et al The need for a complex systems model of evidence for public health. Lancet 2017;390:2602–4. 28622953 10.1016/S0140-6736(17)31267-9

[5] Bonevski B , Randell M, Paul C, et al Reaching the hard-to-reach: a systematic review of strategies for improving health and medical research with socially disadvantaged groups. BMC Med Res Methodol 2014;14:42. 24669751 10.1186/1471-2288-14-42 PMC3974746

[6] McKenzie M , Tulsky JP, Long HL, et al Tracking and follow-up of marginalized populations: a review. J Health Care Poor Underserved 1999;10:409–29. 10581885 10.1353/hpu.2010.0697

[7] David MC , Alati R, Ware RS, Kinner SA. Attrition in a longitudinal study with hard-to-reach participants was reduced by ongoing contact. J Clin Epidemiol 2013;66:575–81. 23384589 10.1016/j.jclinepi.2012.12.002

[8] Culhane DP. The potential of linked administrative data for advancing homelessness research and policy. Eur J Homelessness 2016;10:109–26.

[9] Bramley G , Fitzpatrick S, Wood J, et al Hard Edges Scotland: New Conversations About Severe and Multiple Disadvantage. London: Heriot-Watt University, Lankelly Chase, The Robertson Trust, 2019.

[10] Tweed EJ , Thomson RM, Lewer D, et al Health of people experiencing co-occurring homelessness, imprisonment, substance use, sex work and/or severe mental illness in high-income countries: a systematic review and meta-analysis. J Epidemiol Community Health 2021;75:1010–8. 33893182 10.1136/jech-2020-215975 PMC8458085

[11] Aldridge RW , Story A, Hwang SW, et al Morbidity and mortality in homeless individuals, prisoners, sex workers, and individuals with substance use disorders in high-income countries: a systematic review and meta-analysis. Lancet 2018;391:241–50. 29137869 10.1016/S0140-6736(17)31869-X PMC5803132

[12] Bramley G , Fitzpatrick S, Sosenko F. Mapping the “hard edges” of disadvantage in England: adults involved in homelessness, substance misuse, and offending. Geogr J 2020;186:390–402.

[13] Page A. Turning the tide: a vision paper for multiple needs and exclusions. Advances in Dual Diagnosis 2011;4:173–9.

[14] Harland JM , Adams EA, Boobis S, et al Understanding the life experiences of people with multiple complex needs: peer research in a health needs assessment. Eur J Public Health 2022;32:176–90. 34436575 10.1093/eurpub/ckab142 PMC8975534

[15] Benjaminsen L , Andrade SB. Testing a typology of homelessness across welfare regimes: shelter use in Denmark and the USA. Hous Stud 2015;30:858–76.

[16] Wildeman C , Wang EA. Mass incarceration, public health, and widening inequality in the USA. Lancet 2017;389:1464–74. 28402828 10.1016/S0140-6736(17)30259-3

[17] National Records of Scotland. Population Estimates Time Series Data. Mid-Year Population Estimates: Scotland and Its Council Areas by Single Year of Age and Sex 1981 to 2019. 2020. https://www.nrscotland.gov.uk/statistics-and-data/statistics/statistics-by-theme/population/population-estimates/mid-year-population-estimates/population-estimates-time-series-data (08 January 2021, date last accessed).

[18] Waugh A , Clark A, Knowles J, Rowley D. Health and Homelessness in Scotland. Edinburgh: Scottish Government, 2018.

[19] Morrison DS. Homelessness as an independent risk factor for mortality: results from a retrospective cohort study. Int J Epidemiol 2009;38:877–83. 19304988 10.1093/ije/dyp160

[20] Scottish Government. Provision of HL1 Case-Based Data From 1 April 2007: Guidance Notes. Edinburgh: Scottish Government, 2010.

[21] Graham L , Fischbacher CM, Stockton D, et al Understanding extreme mortality among prisoners: a national cohort study in Scotland using data linkage. Eur J Public Health 2015;25:879–85. 25678604 10.1093/eurpub/cku252

[22] Alvarez-Madrazo S , McTaggart S, Nangle C, et al Data Resource Profile: the Scottish National Prescribing Information System (PIS). Int J Epidemiol 2016;45:714–715f. 27165758 10.1093/ije/dyw060 PMC5005947

[23] Park J , McAlaney C, Connolly M. Improving patient care and clinical governance through the utilisation of a clinical information system. Clin Gov 2008;13:254–60.

[24] Martin JL , McLean G, Park J, et al Impact of socioeconomic deprivation on rate and cause of death in severe mental illness. BMC Psychiatry 2014;14:261. 25227899 10.1186/s12888-014-0261-4 PMC4173101

[25] Scottish Government. Scottish Index of Multiple Deprivation. 2012. https://data.gov.uk/dataset/d9a46acf-ae72-4dc4-b17c-af76222fd6f6/scottish-index-of-multiple-deprivation-simd-20122012 (08 March 2023, date last accessed).

[26] Information Services Division Scotland. Data Dictionary A-Z: CHI Number. 2021. https://www.ndc.scot.nhs.uk/Dictionary-A-Z/Definitions/index.asp?ID=128&Title=CHI%20Number (06 June 2021, date last accessed).

[27] Lex A , Gehlenborg N. Sets and intersections. Nat Methods 2014;11:779.

[28] Homelessness and Rough Sleeping Action Group. Ending Homelessness: The Report on the Final Recommendations of the Homelessness and Rough Sleeping Action Group. Edinburgh: Scottish Government, 2018.

[29] Scottish Drug Deaths Taskforce. Our Work. 2021. https://drugdeathstaskforce.scot/ (12 November 2021, date last accessed).

[30] Richard L , Hwang SW, Forchuk C, et al Validation study of health administrative data algorithms to identify individuals experiencing homelessness and estimate population prevalence of homelessness in Ontario, Canada. BMJ Open 2019;9:e030221. 10.1136/bmjopen-2019-030221 PMC6797366 31594882

[31] Kaushal R , Jagpal P, Khanal S, et al Representation of homeless persons and coding of homelessness in general practices: descriptive evaluation using healthcare utilisation data. BJGP Open 2021;5:BJGPO.2021.0050. 34045292 10.3399/BJGPO.2021.0050 PMC8450878

[32] Wang EA , Long JB, McGinnis KA, et al Measuring exposure to incarceration using the electronic health record. Med Care 2019;57:S157–63. 31095055 10.1097/MLR.0000000000001049 PMC8352066

[33] Gilbert R , Lafferty R, Hagger-Johnson G, et al GUILD: GUidance for Information about Linking Data sets. J Public Health (Oxf) 2018;40:191–8. 28369581 10.1093/pubmed/fdx037 PMC5896589

[34] Metraux S , Culhane DP. Recent incarceration history among a sheltered homeless population. Crime Delinq 2006;52:504–17.

[35] Gisev N , Larney S, Kimber J, et al Determining the impact of opioid substitution therapy upon mortality and recidivism among prisoners: a 22 year data linkage study. Trends Issues Crime Crim Justice 2015;498.

[36] Somers JM , Moniruzzaman A, Rezansoff SN, et al The prevalence and geographic distribution of complex co-occurring disorders: a population study. Epidemiol Psychiatr Sci 2016;25:267–77. 25989819 10.1017/S2045796015000347 PMC6998736

[37] Rezansoff SN , Moniruzzaman A, Gress C, Somers JM. Psychiatric diagnoses and multiyear criminal recidivism in a Canadian Provincial Offender Population. Psychol Public Policy Law 2013;19:443–53.

[38] Downs JM , Ford T, Stewart R, et al An approach to linking education, social care and electronic health records for children and young people in South London: a linkage study of child and adolescent mental health service data. BMJ Open 2019;9:e024355. 10.1136/bmjopen-2018-024355 PMC6352796 30700480

[39] Harron KL , Doidge JC, Knight HE, et al A guide to evaluating linkage quality for the analysis of linked data. Int J Epidemiol 2017;46:1699–710. 29025131 10.1093/ije/dyx177 PMC5837697

[40] Walsh D , McCartney G, Collins C, et al History, Politics and Vulnerability: Explaining Excess Mortality. Glasgow: Glasgow Centre for Population Health, 2016. 10.1016/j.puhe.2017.05.016 28697372

