# Supplementary material for: Co-occurring homelessness, justice involvement, opioid dependence and psychosis: a cross-sectoral data linkage study
Source: Eur J Public Health. 2023 Mar 15;33(2):249–56. doi: 10.1093/eurpub/ckad034 (PMC10066485; doi:10.1093/eurpub/ckad034)
Supplement: ckad034_Supplementary_Data [file ckad034_supplementary_data.docx]

**Supplementary material**

**Section S1. Additional details for source datasets.**

**Table S1.1. Description of data sources used in cohort creation to ascertain experiences of interest**

| **Experience** | **Definition** | **Data source** | **Data collection** | **Selection process**  **(if any)** | **Data provider** |
| --- | --- | --- | --- | --- | --- |
| Homelessness or housing insecurity (HL) | Assessed by Glasgow City Council as homeless or threatened with homelessness (main applicant only) | HL1 | Face-to-face interview between applicant and housing officer | Individual experiencing homelessness applies to local authority for support as per entitlements under Scottish law | Glasgow City Council |
| Justice involvement  (CUST – any prison record; COMM – court report only) | Resident of Glasgow City having previously been received into a Scottish prison | PR2 | Reception process when individual arrives into prison | None | Scottish Prison Service/Scottish Government |
|  | Resident of Glasgow City having been the subject of a submitted criminal justice social work report | Criminal Justice Social Work Reports (CJSWR) | Face-to-face interview between applicant and social work officer | Individual convicted of offence meets statutory criteria for CJSWR or request otherwise made by sheriff | Glasgow City Council |
| Opioid dependence (ODep) | Resident of Glasgow City having received community-dispensed opioid substitution therapy (OST) anywhere in NHSGGC | Prescribing Information System (PIS) | Electronic record of dispensing, generated for reimbursement purposes | Individual with opioid dependence seeks treatment; is prescribed OST (methadone, buprenorphine, or buprenorphine/naloxone); and redeems prescription | NHSGGC |
| Psychosis (PSY) | Resident of Glasgow City with diagnosis of psychotic disorder (excluding psychotic disorder secondary to substance use or the puerperal period) | Glasgow Psychosis Clinical Information System (PsyCIS) | Review of clinical records by research nurse, +/- correspondence with clinical team | Individual experiencing psychosis is in contact with community mental health team | NHSGGC |

**Table S1.2. Definition of Homeless persons and persons threatened with homelessness from 1987 Housing (Scotland) Act, updated in the 2001 Housing (Scotland) Act** (Source: [https://www.legislation.gov.uk/ukpga/1987/26/section/24#commentary-c12770551](https://www.legislation.gov.uk/ukpga/1987/26/section/24%23commentary-c12770551))

| Homeless persons and persons threatened with homelessness:   - A person is homeless if he has no accommodation in the United Kingdom or elsewhere. - A person is to be treated as having no accommodation if there is no accommodation which he, together with any other person who normally resides with him as a member of his family or in circumstances in which the local authority consider it reasonable for that person to reside with him—  1. is entitled to occupy by virtue of an interest in it or by virtue of an order of a court, or 2. has a right or permission, or an implied right or permission to occupy, or in England and Wales has an express or implied licence to occupy, or 3. occupies as a residence by virtue of any enactment or rule of law giving him the right to remain in occupation or restricting the right of any other person to recover possession.   (2A) A person shall not be treated as having accommodation unless it is accommodation which it would be reasonable for him to continue to occupy.  (2B) Regard may be had, in determining whether it would be reasonable for a person to continue to occupy accommodation, to the general circumstances prevailing in relation to housing in the area of the local authority to whom he has applied for accommodation or for assistance in obtaining accommodation.   - A person is also homeless if he has accommodation but—  1. he cannot secure entry to it, or 2. it is probable that occupation of it will lead to abuse (within the meaning of the Protection from Abuse (Scotland) Act 2001 (asp 14)), or  - it is probable that occupation of it will lead to abuse (within the meaning of the Protection from Abuse (Scotland) Act 2001 (asp 14)), from some other person who previously resided with that person, whether in that accommodation or elsewhere, or - it consists of a movable structure, vehicle or vessel designed or adapted for human habitation and there is no place where he is entitled or permitted both to place it and to reside in it; or - it is overcrowded within the meaning of section 135 and may endanger the health of the occupants; or - it is not permanent accommodation, in circumstances where, immediately before the commencement of his occupation of it, a local authority had a duty under section 31(2) in relation to him. - A person is threatened with homelessness if it is likely that he will become homeless within 2 months. - For the purposes of subsection (3)(e), “permanent accommodation” includes accommodation— - of which the person is the heritable proprietor, - secured by a Scottish secure tenancy, - secured by an assured tenancy that is not a short assured tenancy, - where paragraph 1 or 2 of schedule 6 to the Housing (Scotland) Act 2001 (asp 10) is satisfied in relation to the person, secured by a short Scottish secure tenancy. - secured by a private residential tenancy |
| --- |

**Table S1.3. Circumstances whereby a court is required and/or may choose to obtain and consider a report from a local authority officer (that is, a Criminal Justice Social Work Report)**

Source: National Outcomes and Standards for Social Work Services in the Criminal Justice System: Criminal Justice Social Work Reports and Court-Based Services Practice Guidance

| When the offender is a person specified in section 27(1)(b)(i) to (vi) of the Social Work (Scotland) Act 1968, which includes a person who is:  • Under the supervision of a court  • Under supervision or subject to a community service order following release from prison or detention  • Subject to a community service order or a probation order which includes an unpaid work requirement  • Subject to a supervised release order  • Subject to a community reparation order  • Under 16 years of age and subject to a restriction of liberty order  • Aged 16 or 17 years and subject to a supervision requirement  When the court is:  • Passing an extended sentence  • Making a supervised release order [replaced by Community Pay-Back Order*]  • Making a probation order [replaced by Community Pay-Back Order*]  • Making a drug treatment and testing order  • Making a community service order [replaced by Community Pay-Back Order*]  • Considering a custodial sentence for those aged between 16 and 21 years of age  • Considering a custodial sentence for those aged over 21 years of age who have not previously been sentenced to imprisonment or detention in the UK |
| --- |

*****The Community Pay-Back Order replaced probation orders, community service orders, and supervised attendance orders for offences committed after 1 February 2011.

**Table S1.4. ICD-10 codes used to identify individuals with a diagnosis of psychotic disorder in the PsyCIS clinical registry.**

| - Adults aged 18-65 with one of the following ICD-10 diagnoses, diagnosed by a consultant psychiatrist   - F20-29 Schizophrenia, schizotypal and delusional disorder   - F30-F31 Mania and Bipolar Disorder   - F32.3 Severe Depression with psychotic symptoms   - F33.3 Recurrent depressive disorder, current episode severe with psychotic symptoms   - F33.4 Recurrent depressive disorder, currently in remission*   - F06.0 Organic Hallucinosis   - F06.1 Organic catatonic disorder   - F06.2 Organic delusional (schizophrenia- like) disorder   - F06.30 Organic mood (affective) disorders   - F06.31 Organic depressive disorder with psychotic symptoms   - F53.1 Severe mental and behavioural disorders associated with the puerperium, not elsewhere classified   - F1x.5 Mental and behavioural disorders due to psychoactive substance: psychotic disorder   *Where individual has previous diagnosis of psychosis and active ongoing treatment with anti-psychotic medication. |
| --- |

**Section S2. Details of linkage process and success rates**

Linkage process

CHI seeding – the process of matching records from administrative datasets not containing a Community Health Index (CHI) number to the CHI register – was undertaken by the West of Scotland Safe Haven for the HL1, PR2, and CJSWR datasets. The CJSWR dataset already contained CHI numbers for some records, reflecting the incorporation of the criminal justice social work function into Glasgow City Health and Social Care Partnership as part of health and social care integration during the study period.

Before seeding, datasets were checked for consistency in the formatting and length of fields.

CHI seeding was carried out on a deterministic basis supplemented by manual review, using forename, surname, and date of birth OR forename & surname soundex codes combined with date of birth. Soundex codes are anonymised representation of surnames consisting of the initial letter of the surname and three digits, used to enable matching of names despite spelling variations (e.g., Mohammed vs Muhamed, MacDonald vs McDonald). Manual assessments of postcodes were included in supplementary reviews but not in the primary matching process due to migration over time potentially resulting in false negatives.

No reference datasets with known true- and false-matches were available to assess sensitivity or specificity of the CHI seeding process.

Once CHI seeding was complete, all linkages were undertaken on a deterministic basis using CHI numbers.

Results of linkage

The flow charts below describe the linkage process for each of the non-health datasets requiring CHI seeding. CHI seeding was undertaken on the entire datasets provided by the data controllers, which were not restricted to the specific dates of this study: totals for the number of records and of unique individuals may therefore not match those presented in the results for this article.

In contrast to the HL1 and CJSWR datasets, which originated from Glasgow City Health and Social Care Partnership and whose geographical scope therefore fell entirely within the area covered by the West of Scotland Safe Haven, the PR2 (prisons) dataset was national. This allowed us to identify Glasgow City residents who had experienced imprisonment regardless of where in Scotland they had been imprisoned.

**Figure S2.1. Flowchart demonstrating CHI seeding process for HL1 dataset**

**
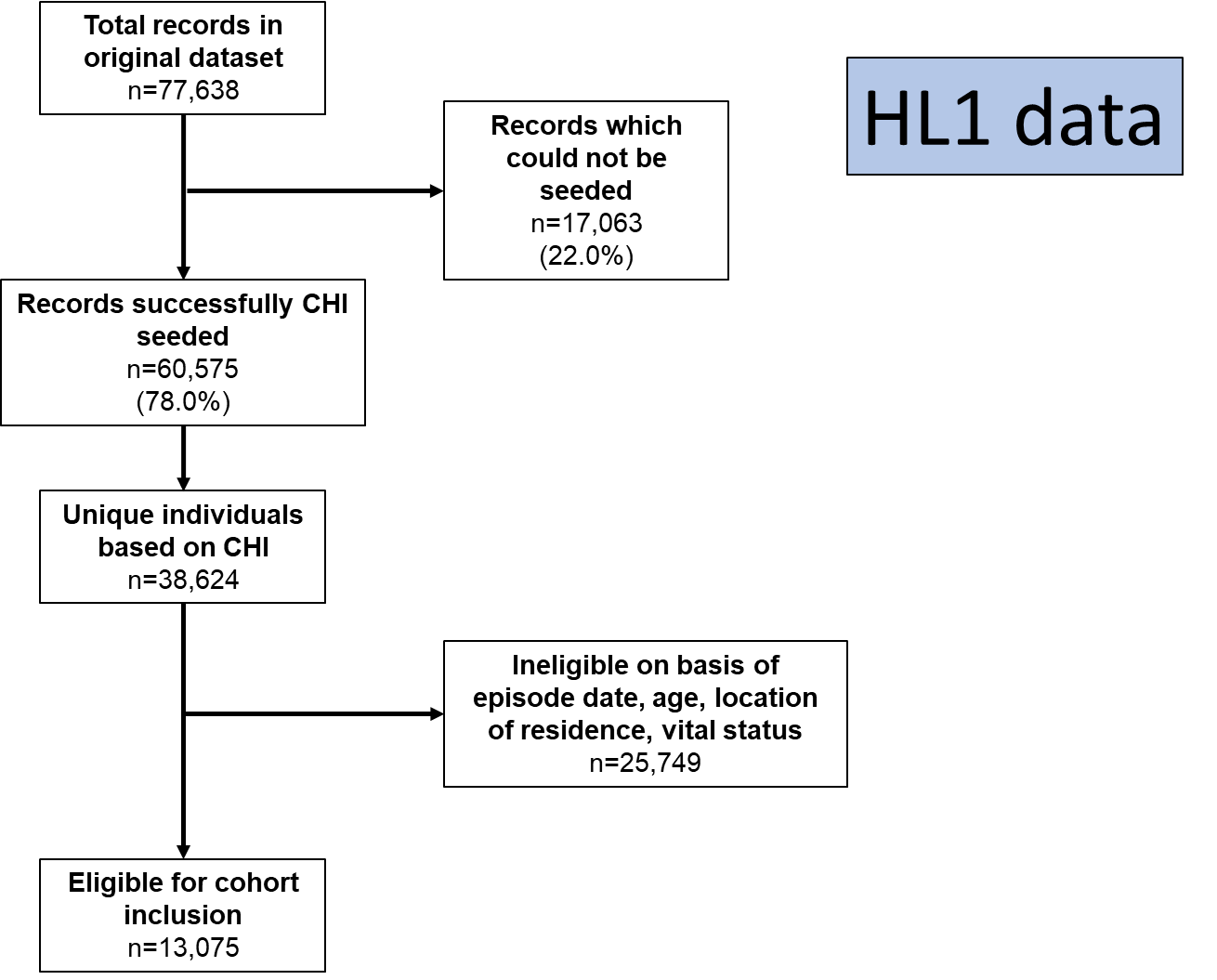
**

**Table S2.1. Comparison of demographic characteristics between records in HL1 dataset which could and could not be assigned a CHI number** (Note that the figures below relate to records, rather than individuals)

| **HL1** | **Able to be assigned a CHI** | |  |
| --- | --- | --- | --- |
|  | **Yes** | **No** | **Total** |
| **Total number of records**  **(%)** | 60,575  (78.0) | 17,063  (22.0) | 77,638  (100.0) |
| **Percentage male** | 57.9 | 52.5 | 57.9 |
| **Mean age** | 44 | 43 | 44 |

**Figure S2.2. Flowchart demonstrating CHI seeding process for CJSWR dataset**

**
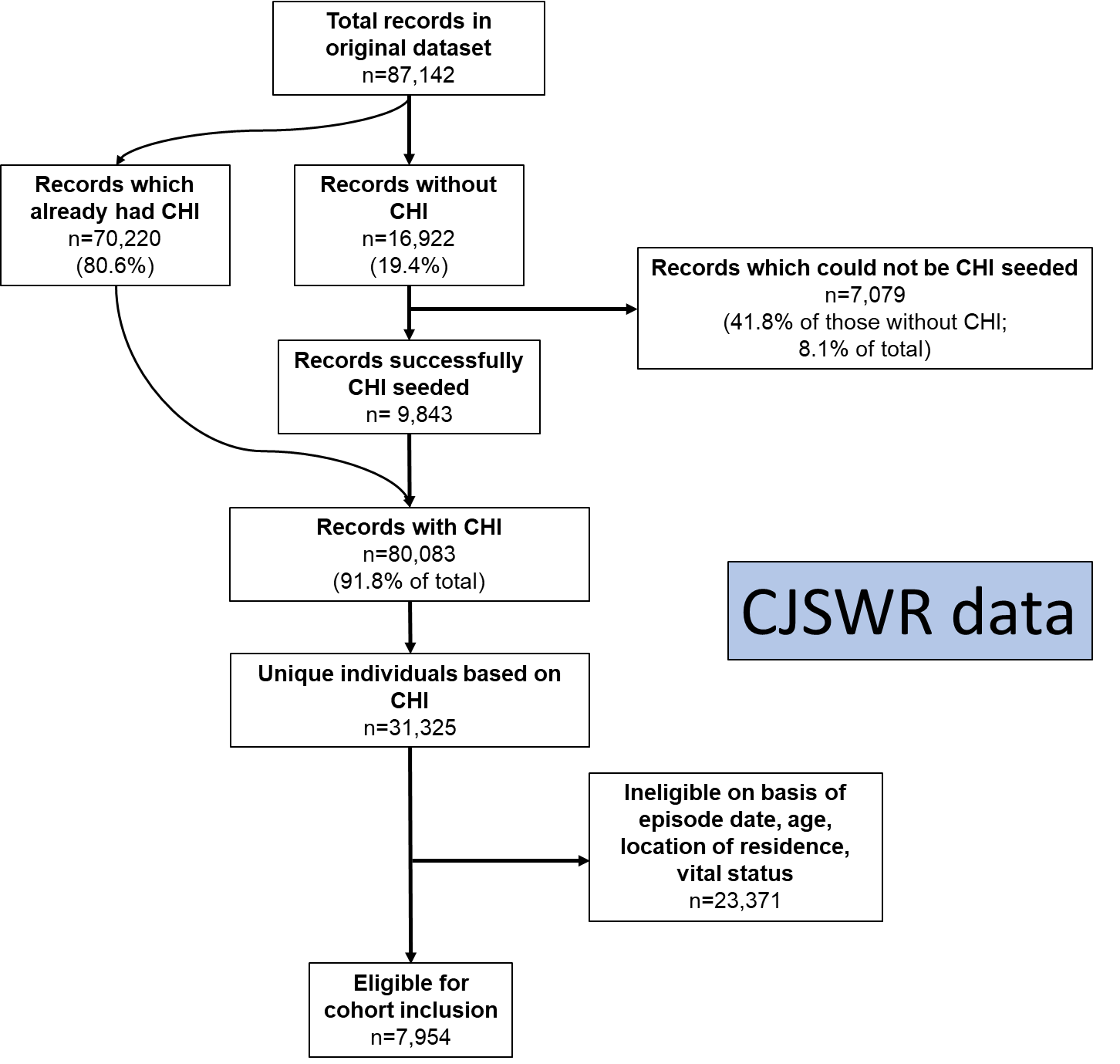
**

**Table S2.2. Comparison of demographic characteristics between records in CJSWR dataset which could and could not be assigned a CHI number**. (Note that the figures below relate to records, rather than individuals; records for whom a CHI number was available in the original CJSWR dataset are included under the ‘Yes’ category)

| **CJSWR** | **Able to be assigned a CHI** | |  |
| --- | --- | --- | --- |
|  | **Yes** | **No** | **Total** |
| **Total number of records**  **(%)** | 80,083  (91.8) | 7,079  (8.2) | 87,142  (100.0) |
| **Percentage male** | 85.4 | 85.0 | 85.4 |
| **Mean age** | 42 | 44 | 42 |

**Figure S2.3. Flowchart demonstrating CHI seeding process for PR2 dataset**

**
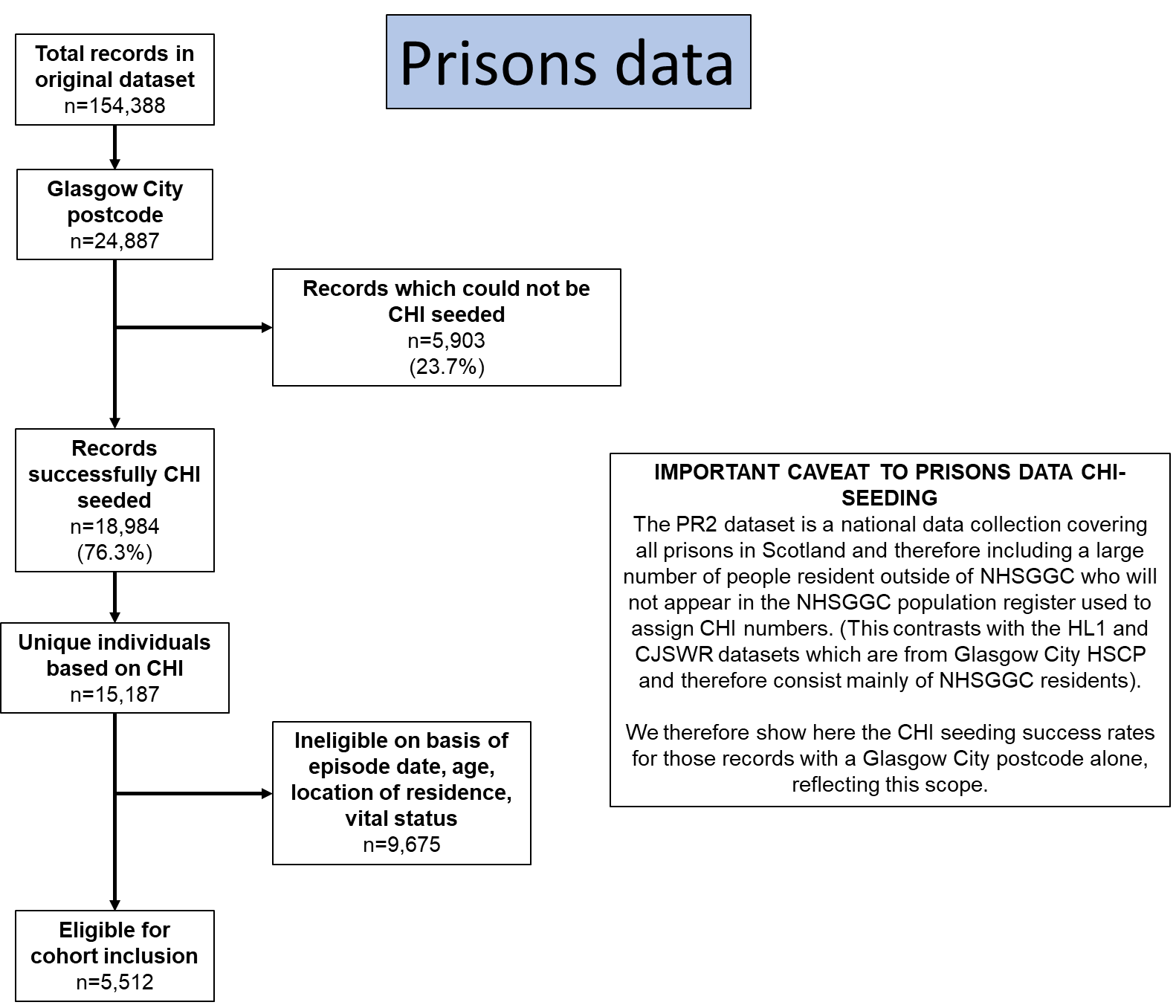
**

**Table S2.3. Comparison of demographic characteristics between records in PR2 dataset which could and could not be assigned a CHI number**. (Note that the figures below relate to records, rather than individuals, and to the overall national dataset from all prisons comprising 154,388 records. Many of those who could not be assigned a CHI number were therefore people resident outside of NHSGGC who will not be included in the NHSGGC population register used to identify CHI numbers.)

| **PR2** | **Able to be assigned a CHI** | |  |
| --- | --- | --- | --- |
|  | **Yes** | **No** | **Total** |
| **Total number of records**  **(%)** | 42,541  (27.6) | 100,926  (72.4) | 154,388  (100.0) |
| **Percentage male** | 92.1 | 90.2 | 90.7 |
| **Mean age** | 43 | 42 | 42 |

**Section S3. Additional results.**

**Table S3.1. Demographic characteristics of adults living in Glasgow City, stratified by exposure to single or multiple experiences of disadvantage, 01/04/2010-31/03/2014**

| **Experience(s) of interest** | **Total**  **(%)** | **% Male**  **(95% CI)** | **Median age**  **(IQR)** | **% most deprived SIMD quintile^§^**  **(95% CI)** |
| --- | --- | --- | --- | --- |
| Total population | 536,653 | 52.6  (52.4 – 52.7) | 40.5  (29.5 – 53.8) | 45.6  (45.5 – 45.7) |
| No experiences of interest | 508,541 | 51.9  (51.7 – 52.0) | 40.7  (29.5 – 54.2) | 44.0  (43.9 – 44.1) |
| Any experience of interest | 28,112 | 64.8  (64.3 – 65.4) | 39.0  (30.5 – 47.6) | 75.2  (74.6 – 75.7) |
|  | | | |  |
| ***Summary of combinations*:*** | | | |  |
| **Any homelessness (HL)** | **13,075** | **54.6**  **(53.7 – 55.4)** | **35.7**  **(28.7 – 45.1)** | **77.8**  **(77.0 – 78.5)** |
| HL only | 9,463 | 46.9  (45.9 – 48.0) | 34.8  (28.1 – 45.6) | 77.4  (76.5 – 78.2) |
| HL + other experience(s) | 3,612 | 74.5  (73.0 – 75.9) | 37.5  (30.8 – 44.3) | 78.9  (77.5 – 80.2) |
| **Any opioid dependence (ODep)** | **7,412** | **68.8**  **(67.7 – 69.8)** | **41.7**  **(36.8 – 46.5)** | **80.3**  **(79.3 – 81.2)** |
| ODep only | 4,123 | 65.3  (63.9 – 66.8) | 42.9  (38.2 – 47.4) | 80.5  (79.2 – 81.7) |
| ODep + other experience(s) | 3,289 | 73.1  (71.5 – 74.6) | 40.0  (35.1 – 45.1) | 80.0  (78.5 – 81.4) |
| **Any justice involvement - custodial (CUST)** | **5,512** | **90.9**  **(90.1 – 91.6)** | **35.6**  **(28.9 – 44.0)** | **76.4**  **(75.1 – 77.5)** |
| CUST only | 2,755 | 94.4  (93.4 – 95.2) | 32.7  (27.0 – 43.0) | 74.0  (72.2 – 75.8) |
| CUST + other experience(s) | 2,757 | 87.4  (86.1 – 88.6) | 37.9  (31.7 – 44.5) | 78.6  (77.0 – 80.2) |
| **Any justice involvement – community (COMM)** | **4,619** | **78.3**  **(77.0 – 79.4)** | **36.4**  **(28.5 – 46.4)** | **73.5**  **(72.2 – 74.8)** |
| COMM only | 3,338 | 81.7  (80.3 – 83.0) | 35.2  (27.8 – 46.9) | 70.6  (69.0 – 72.2) |
| COMM + other experience(s) | 1,281 | 69.4  (66.8 – 71.9) | 38.3  (31.4 – 45.2) | 81.0  (78.7 – 83.2) |
| **Any psychosis (PSY)** | **3,791** | **57.7**  **(56.1 – 59.3)** | **48.6**  **(40.0 – 56.5)** | **63.4**  **(61.8 – 65.0)** |
| PSY only | 3,255 | 55.7  (54.0 – 57.4) | 50.0  (41.5 – 57.7) | 61.2  (59.4 – 62.9) |
| PSY + other experience(s) | 536 | 70.0  (65.9 – 73.8) | 41.7  (34.9 – 48.4) | 77.2  (73.3 – 80.7) |

**^§^** Of those with SIMD data available. SIMD data was available for 96.8% (n=519,757/536,653) of the study cohort.

* Ordered by frequency of ‘any’ category.

**Table S3.2. Number and prevalence of individuals with single versus multiple experiences of disadvantage during the study period, 2010-11 – 2013/14.**

| **Experience of interest** | **This experience only (%)** | **Multiple experiences**  **(%)** | **Total** |
| --- | --- | --- | --- |
| Any homelessness (HL) | 9,463  (72.4) | 3,612  (27.6) | 13,075 |
| Any opioid dependence (ODep) | 4,123  (55.6) | 3,289  (44.4) | 7,412 |
| Any psychosis (PSY) | 3,255  (85.9) | 536  (14.1) | 3,791 |
| Any justice involvement - custodial (CUST) | 2,755  (50.0) | 2,757  (50.0) | 5,512 |
| Any criminal justice social work report (CJSWR) | | | |
| Of which imprisoned (also counted under CUST) | 1,596  (47.9) | 1,739  (52.1) | 3,335 |
| Of which not imprisoned (COMM) | 3,338  (72.3) | 1,281  (27.7) | 4,619 |

Any justice involvement is counted as a single exposure: that is, where individuals have both prison & criminal justice social work report exposures but no other exposures, they will be included in the singly- rather than multiply-exposed category.

**Table S3.3. Number and prevalence of individuals with specific co-occurring exposures during the study period, 2010-11 – 2013/14.**

This table describes the overlap between specific pairs of exposures – it shows, for individuals with the index exposure listed in each row, what number and percentage also had the additional experience listed in the column (with or without any other exposure). For instance, it can be used to identify that 1,825/5,512 (33.1%) of individuals with prison experience during the study period also experienced homelessness during that period (and potentially other exposures as well).

| **Index exposure** | **Total N with this experience**  **(% of total population)** | **Of whom also experienced HL**  **(% of those with**  **index exposure)** | **Of whom also experienced ODep**  **(% of those with**  **index exposure)** | **Of whom also experienced CUST**  **(% of those with**  **index exposure)** | **Of whom also experienced COMM**  **(% of those with**  **index exposure)** | **Of whom also experienced PSY**  **(% of those with**  **index exposure)** |
| --- | --- | --- | --- | --- | --- | --- |
| Any homelessness (HL) | 13,075  (2.4%) | - | 1,840  (14.1%) | 1,825  (14.0%) | 782  (6.0%) | 249  (1.9%) |
| Any opioid dependence (ODep) | 7,412  (1.4%) | 1,840  (24.8%) | - | 1,667  (22.5%) | 641  (8.7%) | 215  (2.9%) |
| Any justice - custodial (CUST) | 5,512  (1.0%) | 1,825  (33.1%) | 1,667  (30.2%) | - | - | 137  (2.5%) |
| Any justice – community only (COMM) | 4,619  (0.9%) | 782  (16.9%) | 641  (13.9%) | - | - | 79  (1.7%) |
| Any psychosis (PSY) | 3,791  (0.7%) | 249  (6.6%) | 215  (5.7%) | 137  (3.6%) | 79  (2.1%) | - |

**Table S3.4. Duration of imprisoned time for those individuals experiencing imprisonment during the exposure period.**

Since imprisonment is the only exposure mutually exclusive with the others, these summary data on total time spent imprisoned (for those individuals experiencing imprisonment during the exposure period) are presented here to inform interpretation of the observed overlap between imprisonment and other exposures, for the primary analysis (1^st^ April 2010 – 31^st^ March 2014).

Of a total 1,460 days in the study period:

|  | Percentage of study period spent imprisoned  (%) | | | | % People imprisoned for:  (n) | |
| --- | --- | --- | --- | --- | --- | --- |
|  | Mean (SD) | Median (IQR) | Min | Max | ≥25% study period | ≥50% study period |
| Total | 16.6  (19.3) | 8.9  (3.0 – 22.9) | 0.0 | 99.6 | 23.0  (1,266) | 7.7  (426) |
| *Summary of multiple vs single exposures:* | | | | |  |  |
| CUST only | 14.9  (19.3) | 7.3  (2.4 – 19.0) | 0.0 | 99.6 | 19.5  (537) | 7.2  (198) |
| CUST + others | 18.2  (19.1) | 11.4  (4.0 – 26.2) | 0.0 | 94.9 | 26.4  (729) | 8.3  (228) |
| *Detailed exposure combinations* | | | | | | |
| CUST only | 14.9  (19.3) | 7.3  (2.4 – 19.0) | 0.0 | 99.6 | 19.5  (537) | 7.2  (198) |
| HL + CUST | 19.6  (19.9) | 12.8  (4.0 – 29.2) | 0.0 | 92.9 | 29.9  (297) | 9.6  (95) |
| ODep + CUST | 15.3  (18.0) | 8.6  (3.4 – 20.2) | 0.1 | 94.9 | 19.3  (163) | 5.7  (48) |
| PSY + CUST | 6.6  (8.0) | 2.7  (0.6 – 9.7) | 0.1 | 35.1 | *≤5 individuals* | *≤5 individuals* |
| HL + ODep + CUST | 21.2  (19.7) | 14.9  (6.0 – 31.1) | 0.1 | 92.2 | 32.3  (252) | 10.8  (84) |
| HL + PSY + CUST | 12.4  (14.1) | 7.3  (1.5 – 15.0) | 0.1 | 57.1 | 20.0  (7) | *≤5 individuals* |
| ODep + PSY + CUST | 11.8  (12.7) | 7.7  (2.6 – 18.2) | 0.1 | 49.9 | *≤5 individuals* | *≤5 individuals* |
| HL + ODep + PSY + CUST | 11.7  (12.3) | 9.5  (2.9 – 13.5) | 0.1 | 46.3 | *≤5 individuals* | *≤5 individuals* |

**Figure S3.1. Distribution of percentage of study period spent imprisoned, by single vs multiple exposures
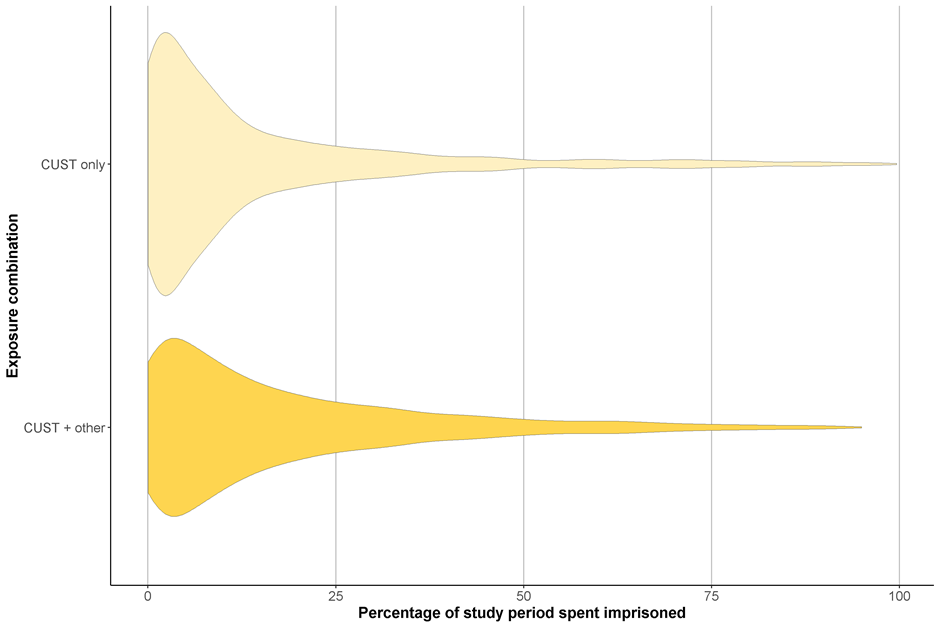
**

**Table S3.5. Ethnicity profile for individuals with experience of homelessness, imprisonment, and courts, 2010/11 – 2013/14.**

Ethnicity was categorised based on information from source datasets using the classification used in Scotland’s Census: <https://www.scotlandscensus.gov.uk/variables-classification/ethnic-group>

The ‘White’ category includes the following options for self-described ethnicity:

White:

- Scottish
- Other British
- Irish
- Gypsy/Traveller
- Polish
- Other white ethnic group, please write in

Ethnicity data was not included in the datasets used to identify individuals with opioid dependence (Prescribing Information System) or to identify the Glasgow City population as a whole (Community Health Index register) so these datasets are not described below, and ethnicity data is not reported for different combinations of experiences.

| Dataset | Experience | Total individuals 2010/11 – 2013/2014 | Number (%) recorded as White ethnicity |
| --- | --- | --- | --- |
| HL1 | Homelessness or housing insecurity | 13,075 | 10,326 (79.0)* |
| PsyCIS | Psychosis diagnosis | 3,791 | 3,462 (91.3) |
| Criminal Justice Social Work Report | Justice involvement – community (+/- custodial) | 7,954 | 7,203 (90.6) |
| PR2 | Justice involvement - custodial | 5,512 | 5,261 (95.5) |

*Note relatively high proportion of those with ethnicity recorded as “Other” – 12% - in the HL1 dataset.

The proportion of the overall Glasgow City population recorded as White ethnicity in the 2011 Census was 88% (UK Data Explorer. Scotland's Census Map 2021 [23/12/2021]. Available from: <https://ukdataexplorer.com/scotland-census-map/>)

**Table S3.6. Number of episodes for selected exposures during the study period, by exposure combination, 2010/11 – 2013/14.**

|  |  | Number of episodes | | | |
| --- | --- | --- | --- | --- | --- |
|  | Number of individuals | Mean  (SD) | Median (IQR) | Min | Max |
| Homelessness | | | | | |
| Any HL | 13,075 | 1.2  (0.6) | 1  (1-1) | 1 | 9 |
| HL only | 9,463 | 1.1  (0.4) | 1  (1-1) | 1 | 8 |
| HL + other exposures | 3,612 | 1.6  (1.0) | 1  (1-2) | 1 | 9 |
|  | | | | | |
| Justice - custodial | | | | | |
| Any CUST | 5,512 | 2.3  (2.1) | 1  (1-3) | 1 | 24 |
| CUST only | 2,755 | 1.8  (1.6) | 1  (1-2) | 1 | 16 |
| CUST + other exposures | 2,757 | 2.8  (2.4) | 2  (1-4) | 1 | 24 |
|  | | | | | |
| Justice – community only | | | | | |
| Any COMM | 4,619 | 1.5  (1.1) | 1  (1-2) | 1 | 16 |
| COMM only | 3,338 | 1.4  (1.0) | 1  (1-1) | 1 | 16 |
| COMM + other exposures | 1,281 | 1.7  (1.3) | 1  (1-2) | 1 | 13 |
|  |  |  |  |  |  |

**Figure S3.2. Number of episodes during study period, among people with experience of these**


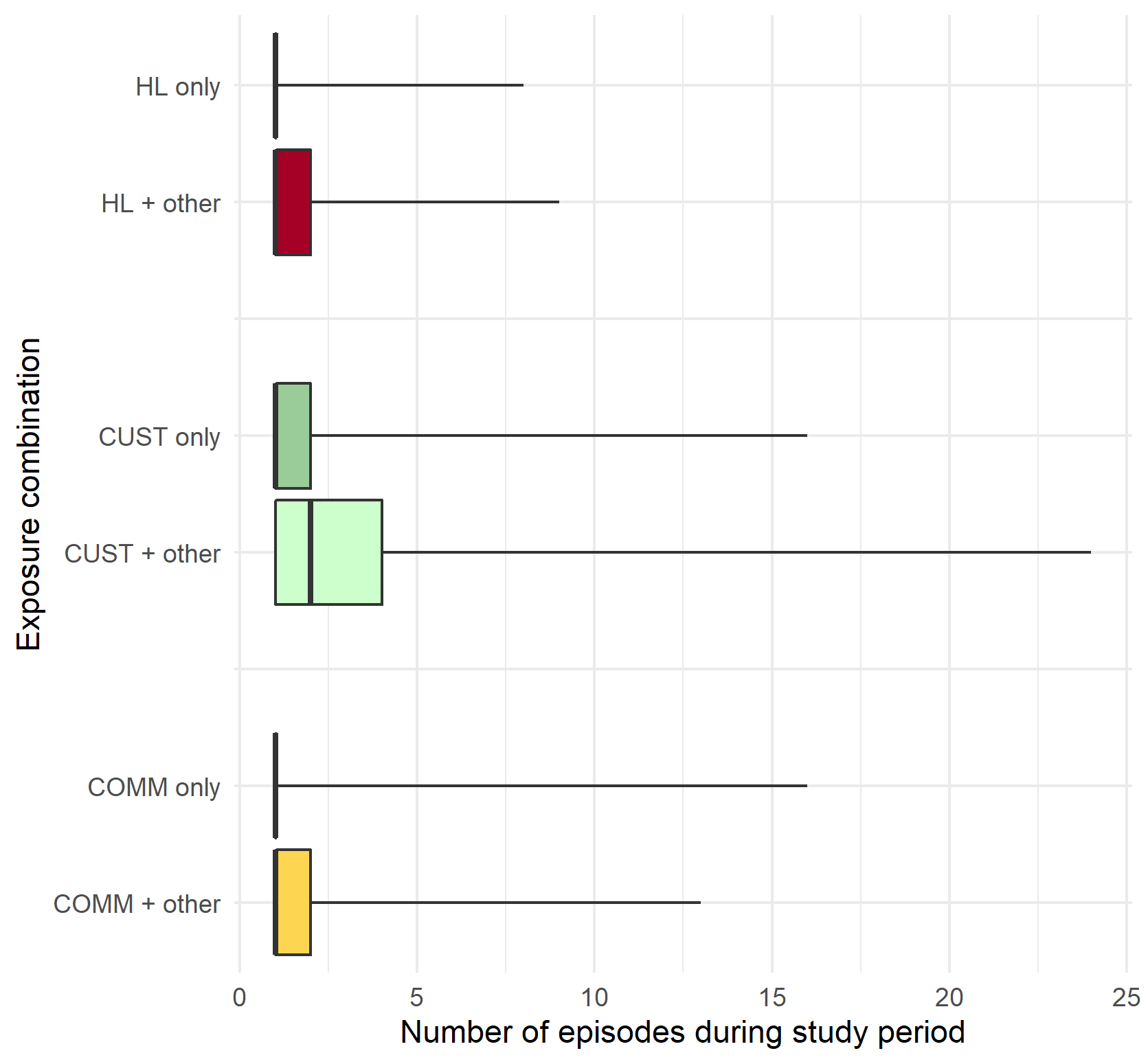


**Table S3.7 – Sensitivity analysis: impact of varying study period length on period prevalence of exposure combinations**

Note that prisons data are only available for the period 2010/11 to 2013/14 so the ‘extended’ study period from 2010/11 to 2015/16 is missing data on imprisonment for the final two years; the results for imprisonment and associated combinations should therefore be interpreted in light of this.

| **Exposure**  **combination*** | **Number (period prevalence, %)** | | |
| --- | --- | --- | --- |
|  | **Study period – primary analyses** | **Study period – restricted** | **Study period - extended** |
|  | **2010/11**  **- 2013/14** | **2012/13**  **- 2013/14** | **2010/11**  **- 2015/16*** |
| Total population | 536,653  (100.0) | 574,092  (100.0) | 507,643  (100.0) |
| No exposures of interest | 508,541  (94.8) | 554,666  (96.6) | 474.701  (93.5) |
| Any exposure of interest | 28,112  (5.2) | 19,426  (3.4) | 32,942  (6.5) |
|  |  |  |  |
| **Summary of exposure combinations:** | | | |
| **Any homelessness (HL)** | **13,075**  **(2.4)** | **6,790**  **(1.2)** | **16,846**  **(3.3)** |
| HL only | 9,463  (1.8) | 5,002  (0.9) | 12,162  (2.4) |
| HL + other exposures | 3,612  (0.7) | 1,788  (0.3) | 4,684  (0.9) |
| **Any opioid dependence (ODep)** | **7,412**  **(1.4)** | **6,743**  **(1.2)** | **7,601**  **(1.5)** |
| ODep only | 4,123  (0.8) | 4,753  (0.8) | 3,792  (0.8) |
| ODep + other exposures | 3,289  (0.6) | 1,990  (0.4) | 3,809  (0.8) |
| **Any justice - custodial (CUST)** | **5,512**  **(1.0)** | **3,598**  **(0.6)** | **5,272**  **(1.0)** |
| CUST only | 2,755  (0.5) | 2,080  (0.4) | 2,364  (0.5) |
| CUST + other exposures | 2,757  (0.5) | 1,518  (0.3) | 2,908  (0.6) |
| **Any justice – community only (COMM)** | **4,619**  **(0.9)** | **2,687**  **(0.5)** | **6,761**  **(1.3)** |
| COMM only | 3,338  (0.6) | 2,077  (0.4) | 4,748  (0.9) |
| COMM + other exposures | 1,281  (0.2) | 610  (0.1) | 2,013  (0.4) |
| **Any psychosis (PSY)** | **3,791**  **(0.7)** | **2,935**  **(0.5)** | **4,281**  **(0.8)** |
| PSY only | 3,255  (0.6) | 2,651  (0.5) | 3,565  (0.7) |
| PSY + other exposures | 536  (0.1) | 284  (0.1) | 716  (0.1) |
| *(continues overleaf)* | | | |

*Listed in order of prevalence during the study period of primary analyses, 2010-2014

**Table S3.7 (continued)**

| **Exposure**  **combination*** | **Number (period prevalence, %)** | | |
| --- | --- | --- | --- |
|  | **2010/11**  **- 2013/14** | **2012/13**  **- 2013/14** | **2010/11**  **- 2015/16*** |
| **Exposures in combination: mutually exclusive category, ordered by frequency**** | | | |
| Homelessness (HL) only | 9,463  (1.8) | 5,002  (0.9) | 12,162  (2.4) |
| Opioid dependence (ODep) only | 4,123  (0.8) | 4,753  (0.8) | 3,792  (0.8) |
| Justice – community (COMM) only | 3,338  (0.6) | 2,077  (0.4) | 4,748  (0.9) |
| Psychosis (PSY) | 3,255  (0.6) | 2,651  (0.5) | 3,565  (0.7) |
| Justice – custodial (CUST) only | 2,755  (0.5) | 2,080  (0.4) | 2,364  (0.5) |
| HL + CUST | 994  (0.2) | 513  (0.1) | 1,096  (0.2) |
| ODep + CUST | 846  (0.2) | 605  (0.1) | 712  (0.1) |
| HL + ODep | 820  (0.2) | 535  (0.1) | 971  (0.2) |
| HL + ODep + CUST | 780  (0.2) | 354  (0.1) | 945  (0.2) |
| HL + COMM | 574  (0.1) | 229  (<0.1) | 952  (0.2) |
| ODep + COMM | 433  (0.1) | 275  (0.1) | 575  (0.1) |
| HL + ODep + COMM | 195  (<0.1) | 68  (<0.1) | 344  (0.1) |
| HL + PSY | 159  (<0.1) | 65  (<0.1) | 229  (0.1) |
| ODep + PSY | 135  (<0.1) | 129  (<0.1) | 151  (<0.1) |
| PSY + CUST | 61  (<0.1) | 26  (<0.1) | 55  (<0.1) |
| PSY + COMM | 56  (<0.1) | 27  (<0.1) | 93  (<0.1) |
| HL + PSY + CUST | 35  (<0.1) | 10  (<0.1) | 52  (<0.1) |
| HL + PSY + ODep | 26  (<0.1) | 6  (<0.1) | 39  (<0.1) |
| ODep + PSY + CUST | 25  (<0.1) | 6  (<0.1) | 26  (<0.1) |
| HL + ODep + PSY + CUST/COMM | 19  (<0.1) | 5  (<0.1) | 31  (<0.1) |
| HL + PSY + COMM | 10  (<0.1) | 3  (<0.1) | 25  (<0.1) |
| OST + PSY + COMM | 10  (<0.1) | 7  (<0.1) | 15  (<0.1) |

*Listed in order of prevalence during the study period of primary analyses, 2010-2014

**Results for HL + ODep + PSY + COMM and HL + ODep + PSY + CUST are grouped here due to small numbers to avoid presenting potentially disclosive information.

**Table S3.8. Sensitivity analysis: impact of varying study period length on prevalence of single versus multiple exposures**

|  | **Multiple exposures -**  **N (%)** | | |
| --- | --- | --- | --- |
| **Exposures of interest** | **2010/11**  **- 2013/14** | **2012/13**  **- 2013/14** | **2010/11**  **- 2015/16*** |
| Any homelessness (HL) | 3,612  (27.6) | 1,788  (26.3) | 4,684  (27.8) |
| Any opioid dependence (ODep) | 3,289  (44.4) | 1,990  (29.5) | 3,809  (50.1) |
| Any prison experience (CUST) | 2,757  (50.0) | 1,518  (42.2) | 2,908  (55.2) |
| Any court report without imprisonment (COMM) | 1,281  (27.7) | 610  (22.7) | 2,013  (29.8) |
| Any psychosis (PSY) | 536  (14.1) | 284  (9.7) | 716  (16.7) |

| **Figure S3.3. Overlap between exposures of interest: sensitivity analyses for change in exposure period.**   1. **Number of individuals with each exposure of interest, and proportion with single vs multiple exposures, by length of study period.** |
| --- |
| **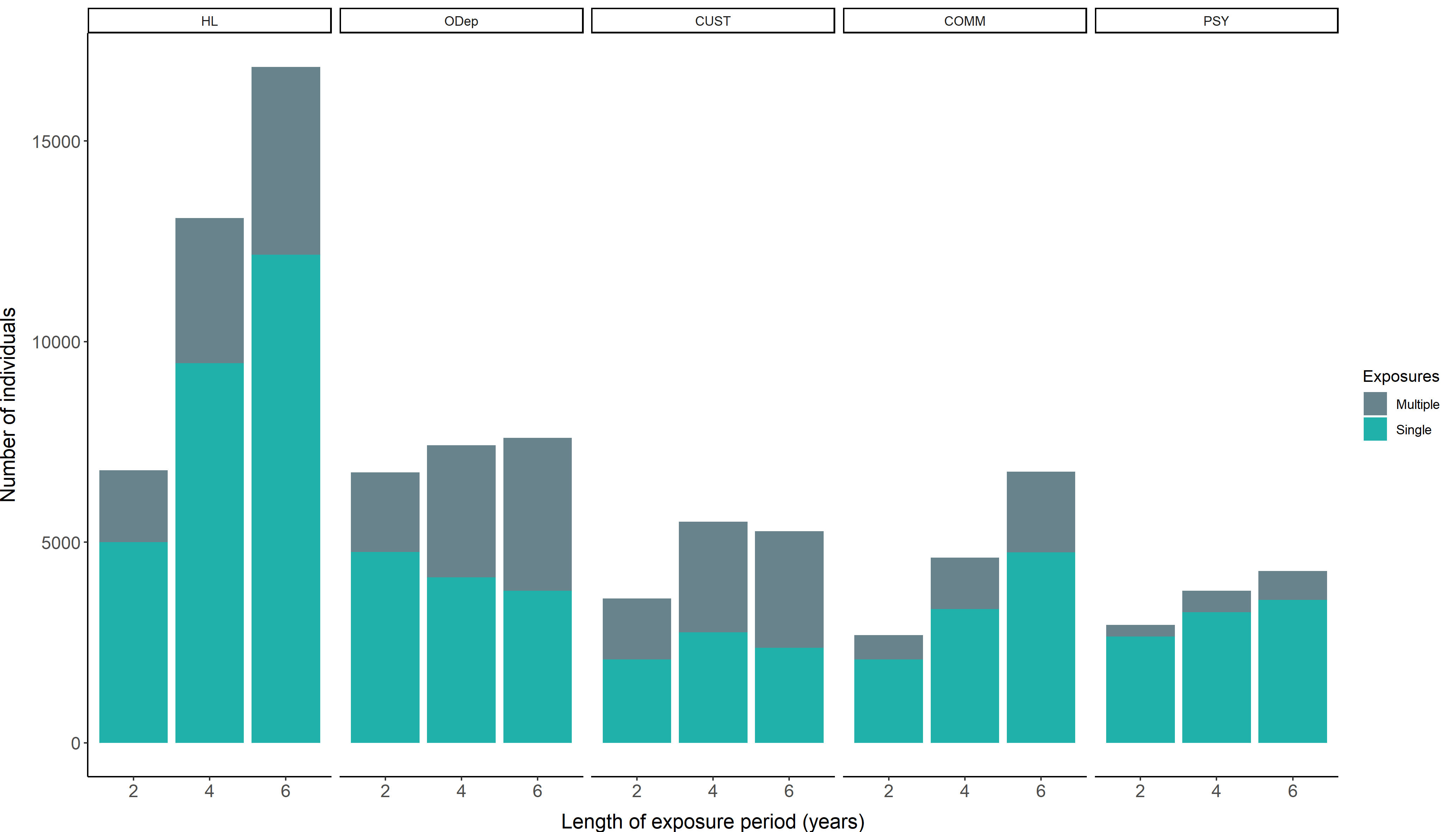** |
| Note that prisons data were only available for the period 2010/11 to 2013/14 so the ‘extended’ study period from 2010/11 to 2015/16 is missing data on imprisonment for the final two years; the results for imprisonment (CUST) should therefore be interpreted in light of this.   1. **Prevalence of individual combinations for the four-year exposure period (primary analysis); 01/04/2010 – 31/03/2014**   **(figure also shown in manuscript; figure 11)** |
| **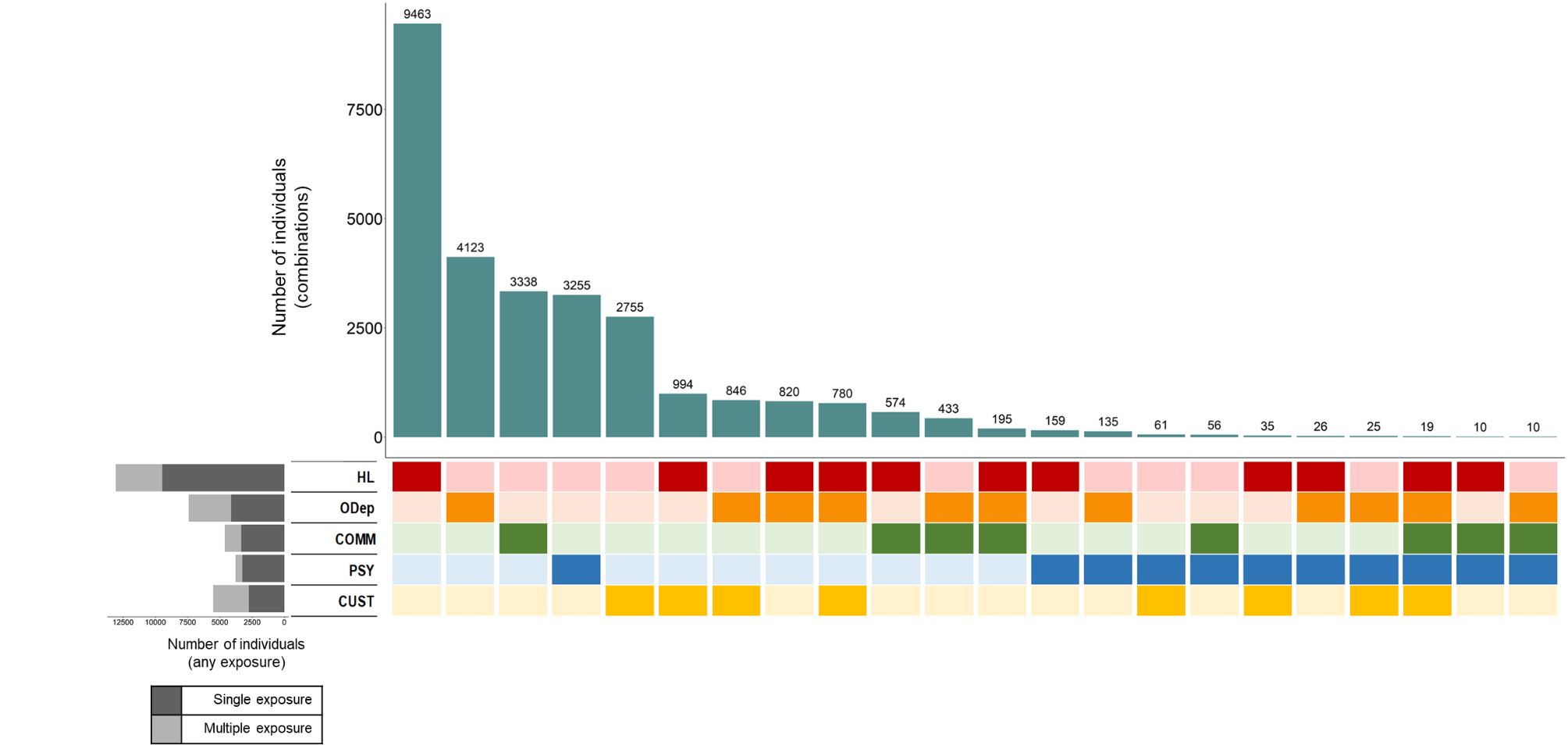** |

Note that exposure combinations are ordered by frequency of mutually exclusive categories.

HL – homelessness and housing insecurity; ODep – opioid dependence indicated by receipt of opioid substitution therapy; COMM – justice involvement in community without imprisonment; PSY – psychosis; CUST – imprisonment.

| 1. **Prevalence of individual combinations for the two-year exposure period; 01/04/2012 – 31/03/2014** |
| --- |
| **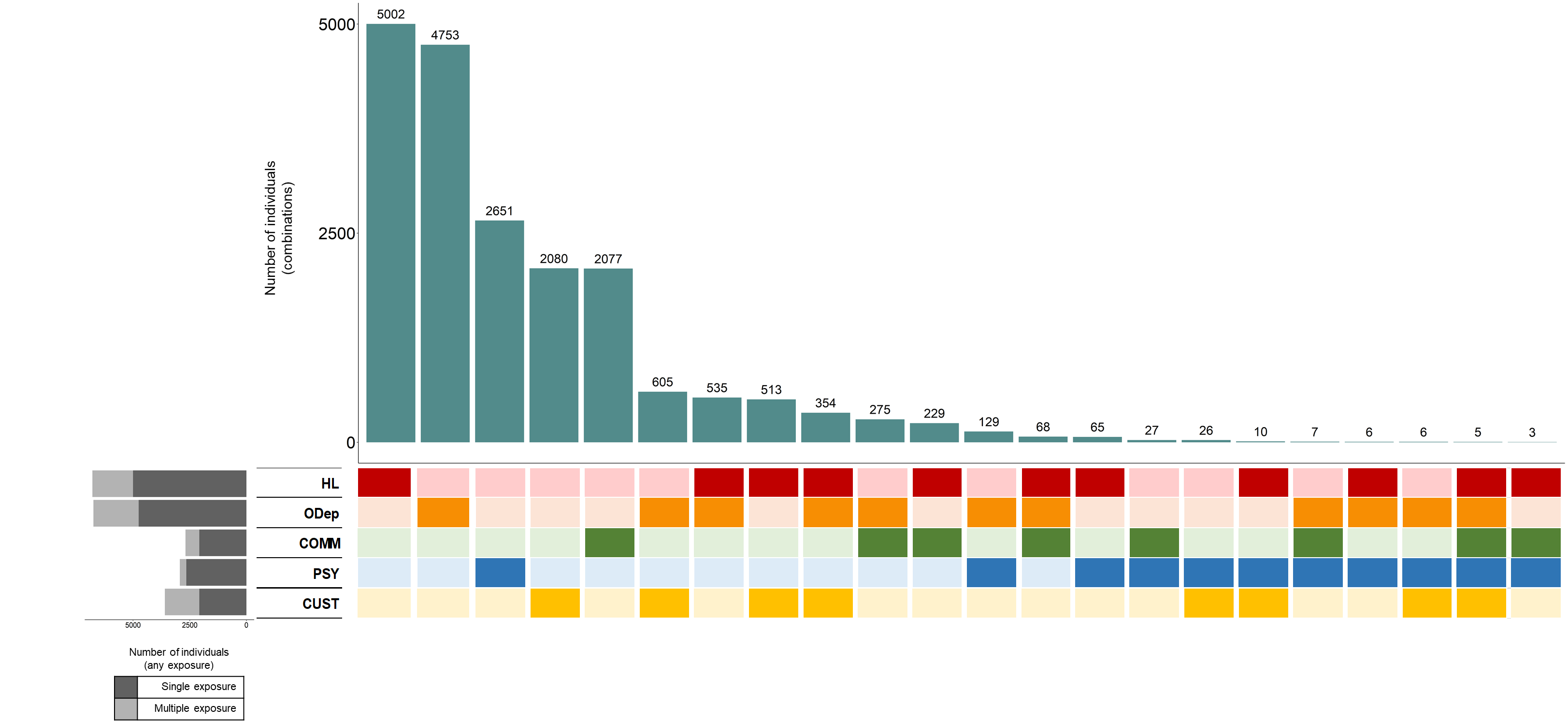** |
| Note that exposure combinations are ordered by frequency of mutually exclusive categories.  HL – homelessness and housing insecurity; ODep – opioid dependence indicated by receipt of opioid substitution therapy; COMM – justice involvement in community without imprisonment; PSY – psychosis; CUST – imprisonment.  **(d) Prevalence of individual combinations for the six-year exposure period; 01/04/2010 – 31/03/2016** |
| **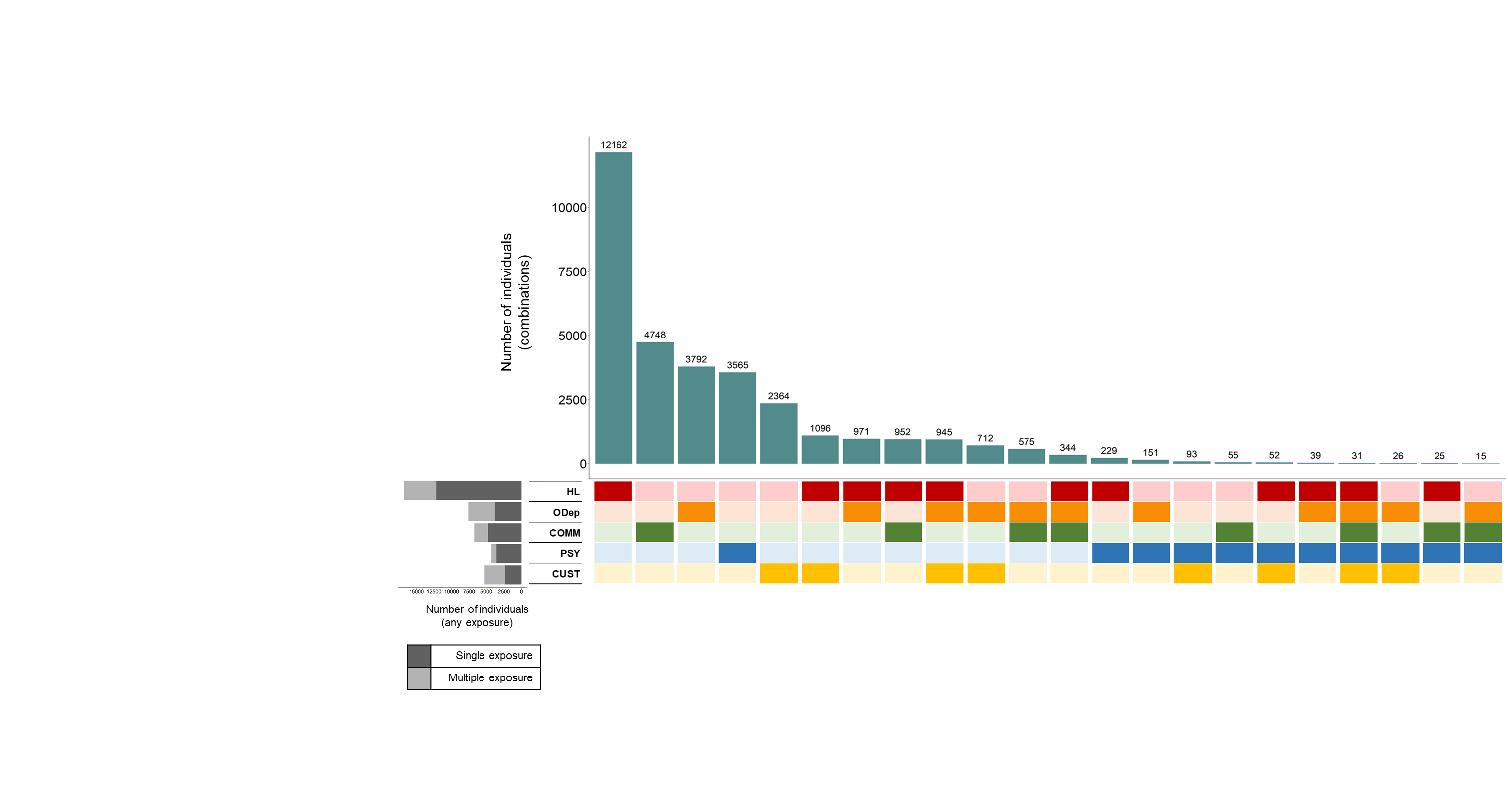** |

Note that exposure combinations are ordered by frequency of mutually exclusive categories as observed in primary analyses, in order to facilitate comparison and interpretation. HL – homelessness and housing insecurity; ODep – opioid dependence indicated by receipt of opioid substitution therapy; COMM – justice involvement in community without imprisonment; PSY – psychosis; CUST – imprisonment.

Prisons data were only available for the period 2010/11 to 2013/14 so the ‘extended’ study period from 2010/11 to 2015/16 is missing data on imprisonment for the final two years; the results for imprisonment (CUST) should therefore be interpreted in light of this.

**Table S3.9. Sensitivity analysis of period prevalence during study period for primary analysis (01/04/2010 – 31/03/2014), comparing use of register-based population denominator and census-based population denominator**

|  | | **Period prevalence**  **(95% CI)** | |
| --- | --- | --- | --- |
| **Exposure**  **combination*** | **Number of individuals** | **Register denominator** | **Census denominator** |
| Homelessness (HL) only | 9,463 | 1.8  (1.8 – 1.8) | 2.1  (2.0 – 2.1) |
| Opioid substitution therapy (ODep) only | 4,123 | 0.8  (0.7 – 0.8) | 0.9  (0.9 – 0.9) |
| Justice – community (COMM) only | 3,338 | 0.6  (0.6 – 0.6) | 0.7  (0.7 – 0.8) |
| Psychosis (PSY) | 3,255 | 0.6  (0.6 – 0.6) | 0.7  (0.7 – 0.7) |
| Justice – custodial (CUST) only | 2,755 | 0.5  (0.5 – 0.5) | 0.6  (0.6 – 0.6) |
| HL + CUST | 994 | 0.2  (0.2 – 0.2) | 0.2  (0.2 – 0.2) |
| ODep + CUST | 846 | 0.2  (0.1 – 0.2) | 0.2  (0.2 – 0.2) |
| HL + ODep | 820 | 0.2  (0.1 – 0.2) | 0.2  (0.2 – 0.2) |
| HL + ODEP + CUST | 780 | 0.2  (0.1 – 0.2) | 0.2  (0.2 – 0.2) |
| HL + COMM | 574 | 0.1  (0.1 – 0.1) | 0.1  (0.1 – 0.1) |
| ODEP + COMM | 433 | 0.1  (0.1 – 0.1) | 0.1  (0.1 – 0.1) |
| HL + ODEP + COMM | 195 | <0.1  (<0.1 - <0.1) | <0.1  (<0.1 - <0.1) |
| HL + PSY | 159 | <0.1  (<0.1 - <0.1) | <0.1  (<0.1 - <0.1) |
| ODEP + PSY | 135 | <0.1  (<0.1 - <0.1) | <0.1  (<0.1 - <0.1) |
| PSY + CUST | 61 | <0.1  (<0.1 - <0.1) | <0.1  (<0.1 - <0.1) |
| PSY + COMM | 56 | <0.1  (<0.1 - <0.1) | <0.1  (<0.1 - <0.1) |
| HL + PSY + CUST | 35 | <0.1  (<0.1 - <0.1) | <0.1  (<0.1 - <0.1) |
| HL + PSY + ODEP | 26 | <0.1  (<0.1 - <0.1) | <0.1  (<0.1 - <0.1) |
| ODEP + PSY + CUST | 25 | <0.1  (<0.1 - <0.1) | <0.1  (<0.1 - <0.1) |
| HL + ODEP + PSY + CUST/COMM | 19 | <0.1  (<0.1 - <0.1) | <0.1  (<0.1 - <0.1) |
| HL + PSY + COMM | 10 | <0.1  (<0.1 - <0.1) | <0.1  (<0.1 - <0.1) |
| ODEP + PSY + COMM | 10 | <0.1  (<0.1 - <0.1) | <0.1  (<0.1 - <0.1) |
| **Total any exposure** | 28,112 | 5.2  (5.2 – 5.3) | 6.2  (6.1 – 6.2) |
| **Total population denominator** | - | 536,653 | 456,237 |

*Listed in order of prevalence during the study period of primary analyses, 2010-2014
